# Chemogenetic regulation of the TARP-lipid interaction mimics LTP and reversibly modifies behavior

**DOI:** 10.1016/j.celrep.2023.112826

**Published:** 2023-07-19

**Authors:** Joongkyu Park, Coralie Berthoux, Erika Hoyos-Ramirez, Lili Shan, Megumi Morimoto-Tomita, Yixiang Wang, Pablo E. Castillo, Susumu Tomita

**Affiliations:** 1 Department of Cellular and Molecular Physiology, Department of Neuroscience, Program in Cellular Neuroscience, Neurodegeneration, and Repair, Kavli Institute for Neuroscience, Yale University School of Medicine, New Haven, CT 06520, USA; 2 Dominick P. Purpura Department of Neuroscience, Albert Einstein College of Medicine, Bronx, NY 10461, USA; 3 Department of Pharmacology, Department of Neurology, Wayne State University School of Medicine, Detroit, MI 48201, USA; 4 Department of Psychiatry and Behavioral Sciences, Albert Einstein College of Medicine, Bronx, NY 10461, USA; 5 These authors contributed equally; 6 Lead contact

## Abstract

Long-term potentiation (LTP), a well-characterized form of synaptic plasticity, is believed to underlie memory formation. Hebbian, postsynaptically expressed LTP requires TARPγ–8 phosphorylation for synaptic insertion of AMPA receptors (AMPARs). However, it is unknown whether TARP-mediated AMPAR insertion alone is sufficient to modify behavior. Here, we report the development of a chemogenetic tool, ExSYTE (Excitatory SYnaptic Transmission modulator by Engineered TARPγ–8), to mimic the cytoplasmic interaction of TARP with the plasma membrane in a doxycycline-dependent manner. We use this tool to examine the specific role of synaptic AMPAR potentiation in amygdala neurons that are activated by fear conditioning. Selective expression of active ExSYTE in these neurons potentiates AMPAR-mediated synaptic transmission in a doxycycline-dependent manner, occludes synaptically induced LTP, and mimics freezing triggered by cued fear conditioning. Thus, chemogenetic controlling of the TARP-membrane interaction is sufficient for LTP-like synaptic AMPAR insertion, which mimics fear conditioning.

## In brief

A chemogenetic tool, ExSYTE, controls the interaction of TARP with plasma membranes and potentiates AMPAR-mediated transmission, thereby mimicking synaptically induced LTP. Selective expression of ExSYTE in activated neurons modifies behavior in a chemogenetic manner.

## Graphical Abstract

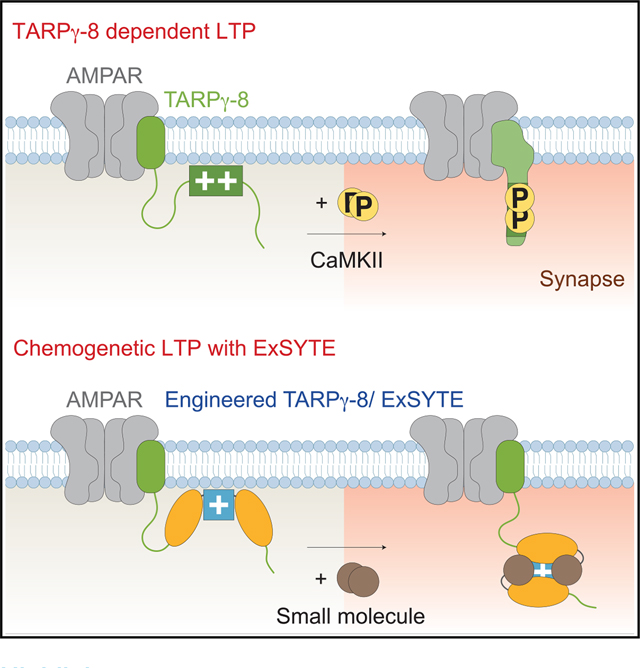

## INTRODUCTION

Long-term potentiation (LTP) of excitatory synaptic transmission is a widely accepted model underlying sustained modifications in neuronal circuits and behaviors.^1–4^ NMDAR-dependent LTP is the most characterized form of long-term strengthening of excitatory synaptic transmission in the brain. Here, calcium influx through NMDARs activates CaMKII (Ca^2+^/calmodulin-dependent protein kinase II), which promotes AMPA receptor (AMPAR) insertion at synapses, thereby increasing AMPAR-mediated transmission.^5,6^ AMPARs interact with the auxiliary subunit TARP (transmembrane AMPAR regulatory protein), which is known to regulate AMPAR localization and channel properties.^7,8^ We recently reported that one of the TARP isoforms, TARPγ–8, is a CaMKII substrate critically involved in NMDAR-mediated LTP.^9^ Relative to wild-type (WT) controls, TARPγ–8 knockin mice carrying non-phosphomimetic mutations at two CaMKII phosphorylation sites exhibit impaired LTP of AMPAR-mediated transmission and performance in both cued and contextual fear conditioning (FC) tests.^9^ The cytoplasmic domain of another TARP family protein TARPγ−2 can interact with negatively charged lipids (i.e., the inner leaflet of plasma membranes), and its phosphorylation dissociates the cytoplasmic domain from the plasma membranes.^10^ However, it remains elusive whether the dissociation of the TARP cytoplasmic domain from the plasma membrane is sufficient for synaptic AMPAR insertion during LTP, neuronal circuit modifications, and behavioral changes.

Long-lasting, activity-dependent changes in synaptic strength at key brain circuits are thought to contribute to learning and memory.^11,12^ A causal link between LTP and learning has been difficult to demonstrate.^1–4^ In the amygdala, a brain area critically involved in fear learning, repetitive activation of auditory inputs with a protocol that induces LTP *in vivo*, can reactivate a fear memory.^13^ However, because repetitive stimulation can trigger multiple forms of LTP and non-synaptic changes in a circuit,^14,15^ it remains unclear whether NMDAR-dependent LTP alone supports fear learning. A tool to induce one form of LTP without neuronal stimulation, for example, a combination of engineered proteins and previously unrecognized small molecules known as chemogenetics, can distinguish specific roles of each form of LTP *in vivo*, though such tools are currently unavailable.

In the present study, we developed a chemogenetic tool to control protein-lipid interactions and incorporated it into the cytoplasmic domain of TARPγ–8 as ExSYTE (Excitatory SYnaptic Transmission modulator by Engineered TARPγ–8) to test whether TARP interaction with lipids is sufficient for potentiating AMPAR-mediated transmission—as it occurs in LTP—and for changing animal behavior using FC as a model. By hijacking the endogenous LTP machinery that mediates AMPAR insertion into synapses, we were able to control the strength of AMPAR-mediated transmission *in vivo*. Using this unique tool, we report that synaptic AMPAR insertion alone in the amygdala is sufficient to modify behavior.

## RESULTS

### Development of a chemogenetic module to control a protein-lipid interaction

**Figure 1.**
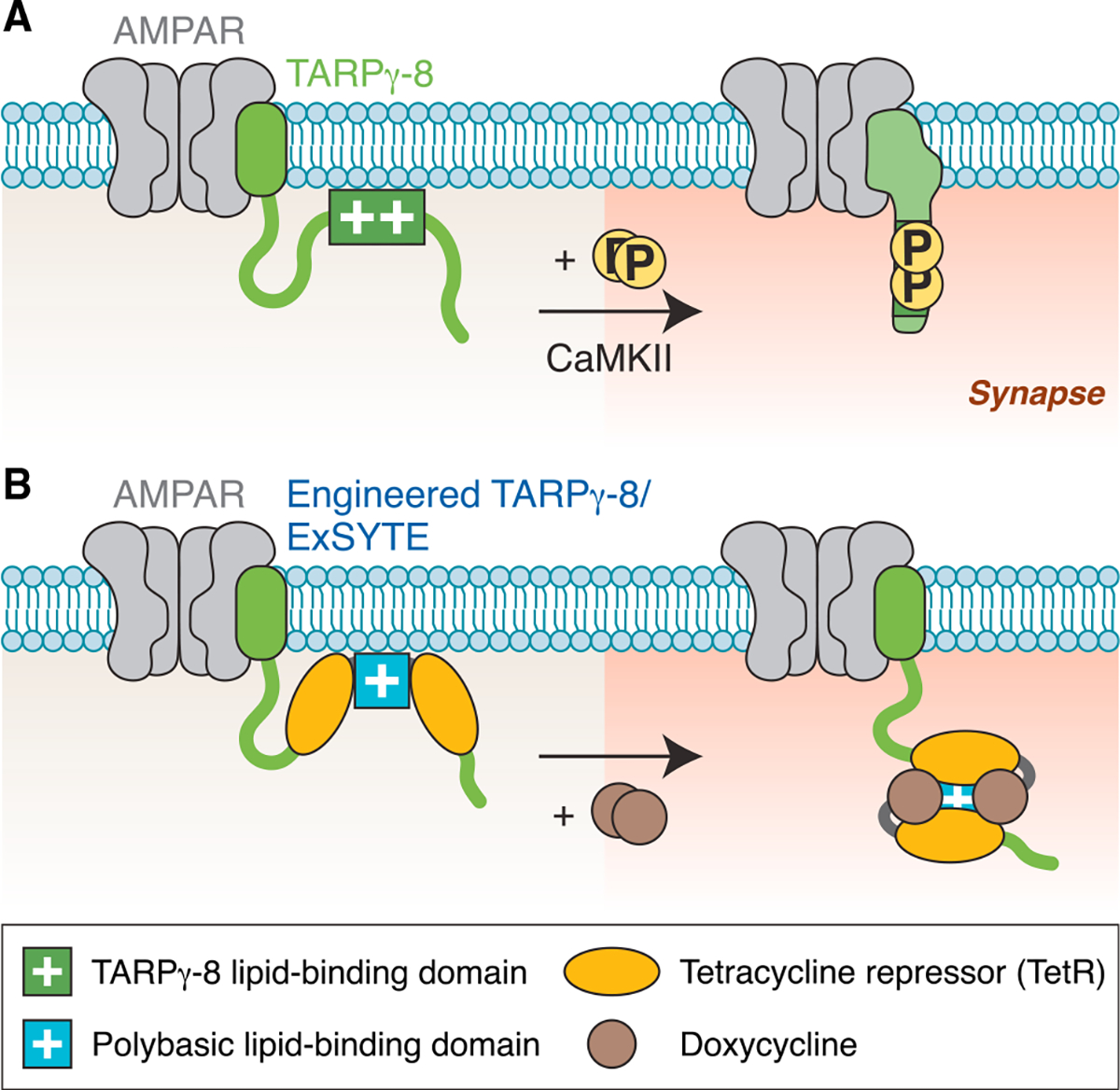
Schematic diagram of a chemogenetic synaptic modulator by engineered TARPγ–8 (A) Model of TARPγ–8-mediated LTP. The TARP cytoplasmic domain contains polybasic residues that bind to negatively charged membranes. Upon CaMKII-mediated TARPγ–8 phosphorylation, the phosphorylated TARP cytoplasmic domain dissociates from the membrane and increases synaptic AMPAR activity. (B) Design of a controllable TARP mutant responsive to doxycycline. To control TARP interaction with the plasma membrane, the TARP cytoplasmic domain was modified by the insertion of a chemogenetic module consisting of a polybasic lipid-binding domain and doxycycline-binding tetracycline repressors.

TARPs have a positively charged arginine-rich cytoplasmic domain that interacts with negatively charged plasma membranes, and this lipid-binding domain (LBD) dissociates from the plasma membrane upon phosphorylation, resulting in increased synaptic AMPAR-mediated transmission at cerebellar mossy fiber-granule cell synapses.^10^ Given that TARPγ–8 is a critical CaMKII substrate of NMDAR-dependent LTP,^9^ we hypothesized that controlling the membrane association of the TARPγ–8 cytoplasmic LBD could provide a simple mechanism to control AMPAR-mediated transmission and mimic synaptically induced LTP in the absence of neuronal stimulation (Figure 1A). We further set out to make the TARPγ–8 cytoplasmic domain-plasma membrane interaction regulatable by adding a small molecule such as doxycycline (Dox) (Figure 1B), which has no obvious effects by itself on mouse behavior.^16–18^ To mimic the phosphorylation-dependent TARP interaction with the plasma membrane, we first developed a cytosolic module that binds to and dissociates from the plasma membrane in a Dox-dependent manner. The efficiency of this module was assessed by examining the localization of enhanced green fluorescent protein (EGFP)-fused proteins expressed in HeLa cells with spinning disk confocal microscopy. We fused a Dox-binding tetracycline repressor protein (TetR)^19^ to EGFP at the TetR N terminus, and this protein did not localize to the plasma membrane (Figures S1A and S1B). We added a polybasic LBD derived from K-ras (KRϕ)^20,21^ between EGFP and TetR (Figure S1A) because KR0ϕ consists of basic residues similar to the TARPγ–8 LBD, but lacks phosphorylation sites, thereby preventing any contribution of endogenous kinases. This protein localized to the plasma membrane but did not dissociate from the membrane in response to Dox administration (Figures S1A and S1B). Given the structure of TetR dimers induced by Dox binding,^22^ we put two TetRs beside the LBD such that Dox-induced dimerization of two TetRs could mask the protein-lipid interaction (Figure S1C). This TetR-KRϕ-TetR peptide localized at the plasma membranes, and Dox addition resulted in limited relocation from the plasma membrane to the cytoplasm (Figures S1A and S1B). We optimized the length and composition of the LBD to improve the efficiency of both the LBD-membrane dissociation (Figures S1A and S1B) and the expression of the peptide by replacing TetRs with codon-optimized TetRs (oTetRs) (Figures S1A and S1D). The LBD and the Arg-rich region of TARPγ–8 fused with two TetRs showed less effective localization to the plasma membranes (Figures S1E and S1F).

**Figure 2.**
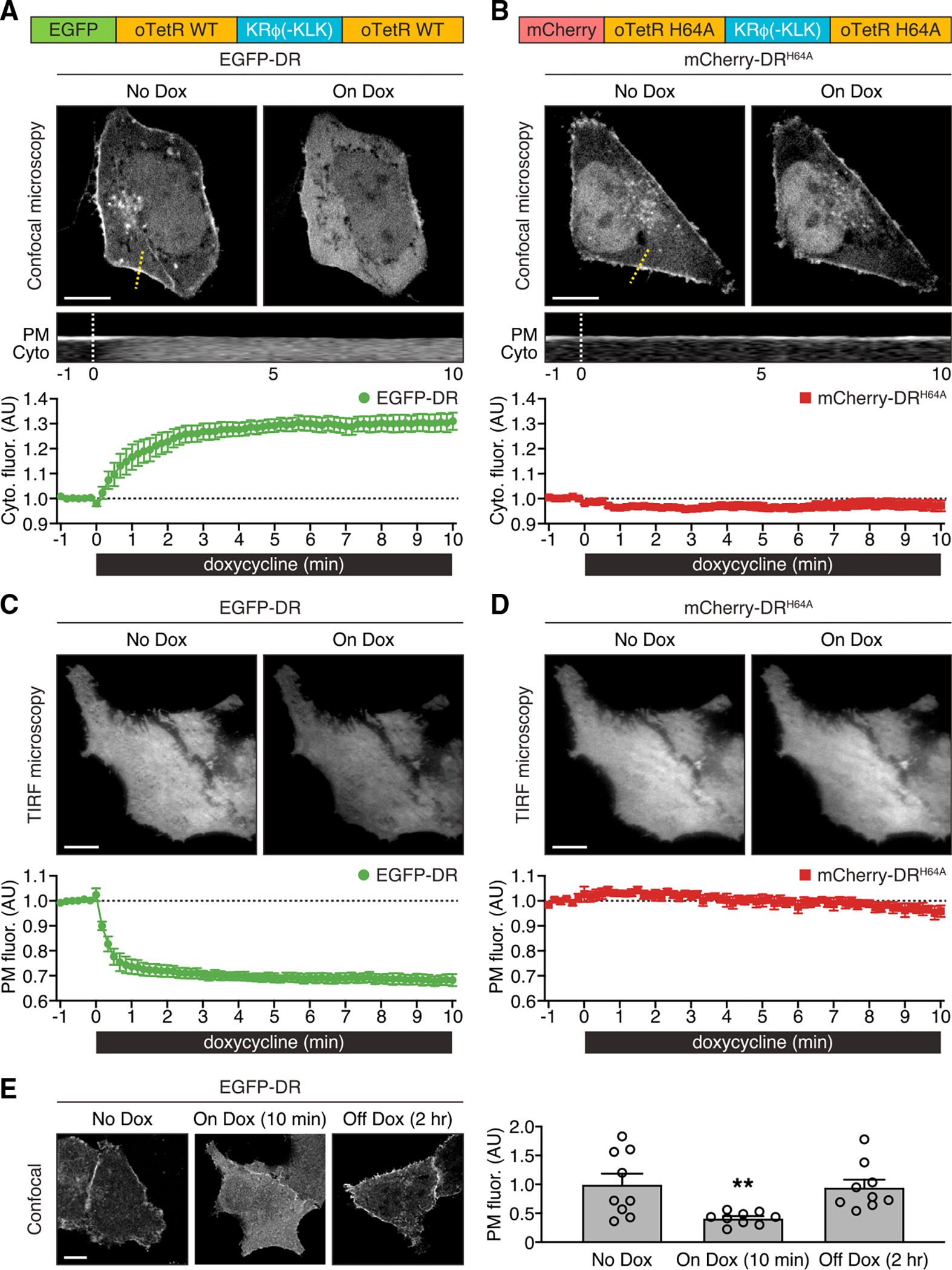
Development of a chemogenetic lipid-binding module The DR consists of a polybasic lipid-binding domain modified from K-ras (KRϕ-KLK) surrounded by oTetRs. (A–D) Distribution of EGFP-fused DR and mCherry-fused DR mutant (H64A) was examined in transiently transfected HeLa cells by spinning-disc confocal (A and B) and TIRF (C and D) microscopies. Both EGFP-DR (A and C) and mCherry-DR^H64A^ (B and D) localized at the plasma membrane (No Dox). Ten minutes after 10 μM Dox treatment (On Dox), EGFP-DR, but not mCherry-DR^H64A^, showed increased cytoplasmic signal (A and B) and reduced TIRF signal from the proximal plasma membrane (C and D). Representative images (top), kymographs (middle; from the dashed yellow lines in A and B) and quantification (bottom) of cytoplasmic fluorescence (A and B; n = 6 cells) and proximal plasma membrane fluorescence (C and D; n = 8 cells). (E) Reversible relocation of EGFP-DR. EGFP-DR localization was examined in different cells with various treatments. (Left) Representative images showing that 2 h after Dox removal from media (Off Dox), EGFP-DR localized to the plasma membranes, similar to that seen in the absence of Dox (No Dox). In contrast, EGFP-DR treated with Dox for 10 min (On Dox) dissociated from the plasma membranes. (Right) Summary plots of the plasma membrane (PM)-associated EGFP-DR fluorescence (n = 9 cells). Scale bar, 10 μm. Data are presented as mean ± SEM. **p < 0.01, one-way ANOVA followed by post hoc Tukey’s multiple comparisons.

Finally, to chemogenetically control the localization of the peptide to the plasma membrane, we developed a cytosolic Dox-responsive module (DR) and a negative control mutant produced by substituting the histidine at residue 64 with alanine (H64A) in each oTetR to disrupt Dox-binding^22^ (Figures 2A and 2B). Both EGFP-DR and mCherry-DR^H64A^ localized mostly at the plasma membrane and weakly on intracellular vesicular and nuclear membranes (Figures 2A and 2B). Dox addition increased EGFP-DR signal levels in the cytoplasm (t_50_ = 1.1 ± 0.3 min; the time required to reach 50% completion) (Figure 2A). In contrast, mCherry-DR^H64A^ as well as EGFP-DR^H64A^ did not respond to Dox (Figures 2B, S2A, and S2B). Total internal reflection fluorescence (TIRF) microscopy was performed to further confirm membrane localization demonstrated a reduction in EGFP-DR signal upon Dox application with a 0.6 ± 0.1 min t_50_, but no change in mCherry-DR^H64A^ localization (Figures 2C, 2D, S2C, and S2D). Furthermore, the plasma membrane localization of EGFP-DR was restored within 2 h of Dox removal from the media (Figure 2E), indicating that the Dox-dependent relocation of the DR module is reversible. These results demonstrate the development of a chemogenetic cytosolic module whose membrane binding can be controlled by Dox.

### A chemogenetic tool ExSYTE for postsynaptically enhancing excitatory transmission

**Figure 3.**
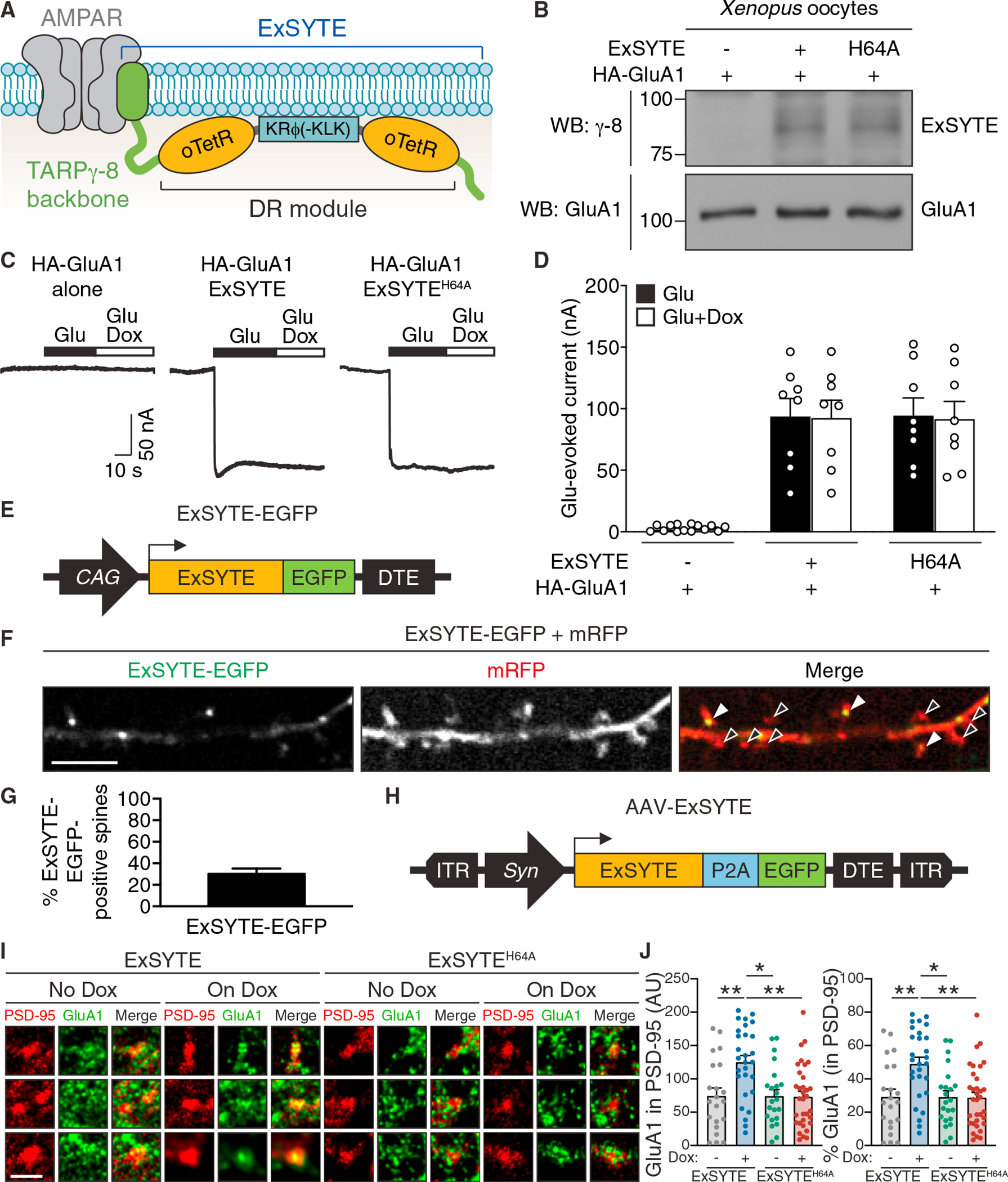
ExSYTE as a chemogenetic modulator of excitatory postsynaptic strength (A) The cytoplasmic domain of TARPγ–8 (residues 257–357) was replaced with DR or DR^H64A^ and referred to as Excitatory SYnaptic Transmission modulator by Engineered TARPγ–8 (ExSYTE) and ExSYTE^H64A^. (B–D) cRNAs of HA epitope-tagged GluA1 (HA-GluA1) and ExSYTE (or ExSYTE^H64A^) were injected into *Xenopus laevis* oocytes. SDS-PAGE showed that ExSYTE and ExSYTE^H64A^ were expressed at similar levels (B). Glutamate (Glu)-evoked currents (100 μM) were monitored using two-electrode voltage-clamp recording with or without 10 μM Dox (C and D). No obvious changes in AMPAR activity were observed between ExSYTE and ExSYTE^H64A^ with or without Dox. Representative traces (C) and summary plots are shown (D; n = 8 oocytes). (E) An *Arc* mRNA DTE was inserted in the ExSYTE construct fused with EGFP to enrich expression in activated dendritic segments. (F and G) The ExSYTE-EGFP and mRFP constructs were co-transfected into primary hippocampal neurons at DIV16, and cells were imaged the next day. Representative images (F) showed ExSYTE-EGFP (green) was detected in some spines (closed arrow), whereas most spines were labeled with mRFP (red) (open arrows). Scale bar, 10 μm. Summary plot (G) represents the fraction of ExSYTE-EGFP-positive spines relative to mRFP^+^ spines (n = 22 dendrites from five cells). (H) Schematic diagram illustrating the AAV-ExSYTE. (I and J) Colocalization of endogenous GluA1 and PSD-95 was examined in cultured hippocampal neurons expressing either ExSYTE or ExSYTE^H64A^ in the absence or presence of doxycycline (Dox) using the Leica SP8 Gated STED 3× super-resolution microscope. The colocalization of GluA1 (green) with PSD-95 (red) increased upon 10 μM Dox treatment for 20 min in neurons expressing ExSYTE, but not the inactive ExSYTE^H64A^ (n = 21–33 images per group). Three representative STED micrographs are shown in (I) and the quantification of GluA1 colocalization in the PSD-95 region is shown in (J). Scale bar, 1 μm. Data are shown as mean ± SEM. *p < 0.05, **p < 0.01, Kruskal-Wallis tests.

To examine whether controlling the interaction of the TARP cytoplasmic domain with the plasma membrane can modulate synaptic AMPAR activity, we replaced a part of the TARPγ–8 cytoplasmic domain (residues 257–357), including the LBD, with the DR or DR^H64A^ module and called these engineered TARPs ExSYTE and ExSYTE^H64A^ (Figure 3A). Both ExSYTE and ExSYTE^H64A^ were expressed at similar levels at the expected molecular weight of 83 kDa in *Xenopus laevis* oocytes co-injected with extracellularly HA-tagged GluA1 on sodium dodecyl sulfate-polyacrylamide gel electrophoresis (SDS-PAGE), and neither ExSYTE nor ExSYTE^H64A^ affected HA-tagged GluA1 expression (Figure 3B).

TARPs increase glutamate-evoked current through AMPARs by modulating surface expression and channel properties of AMPARs.^23^ We therefore examined the modulatory effects of ExSYTE on AMPAR-mediated currents. Whereas no glutamate-evoked currents were detected in oocytes injected with a minimal amount of HA-GluA1 cRNA (0.1 ng), as shown previously,^23^ robust glutamate-evoked currents of similar magnitudes were detected in oocytes co-injected with cRNAs of HA-GluA1 and either ExSYTE or ExSYTE^H64A^ (Figures 3C and 3D). Dox addition did not affect these glutamate-evoked currents (Figure 3D), suggesting that Dox did not alter the channel activity and conductance of the ExSYTE-AMPAR complex. In addition, using the cRNA-injected oocytes, we examined the effect of N-terminally and C-terminally EGFP-tagged ExSYTE (EGFP-ExSYTE and ExSYTE-EGFP) on AMPAR function. We found that both ExSYTE and ExSYTE-EGFP enhanced glutamate-evoked currents, whereas EGFP-ExSYTE did not (Figure S3A).

To enrich ExSYTE expression in activated dendritic segments, we used a dendritic targeting element (DTE) from rat *Arc* mRNA, which is expected to be targeted to dendrites and translated in activated dendritic segments.^24–26^ The ExSYTE construct blocks the PDZ binding site by the addition of EGFP or P2A peptide (Figure 3E). Because the TARPγ–8Δ4 knockin mice lacking the C-terminal PDZ binding site showed normal LTP,^27^ it is unlikely that blocking the PDZ domain binding site would affect the role of TARPγ–8 in LTP. To examine ExSYTE localization in neurons, ExSYTE was fused with EGFP at its C terminus (Figure 3E) and co-transfected with either monomeric red fluorescent protein (mRFP) or near-infrared protein (iRFP)^28^ into primary hippocampal neurons. ExSYTE-EGFP signals from a construct without DTE were detected diffusely on dendrites and spines similar to iRFP distribution, whereas ExSYTE-EGFP signals from a construct with DTE were detected distinctly as puncta (Figures S3B and S3C). Of note, endogenous TARPγ–8 also localizes on spines as well as dendrites (Figure S3D). ExSYTE-EGFP signals were detected mostly on dendritic spines (30.2% ± 5.0% of spines) and neurites (Figures 3F and 3G), suggesting that its expression was limited to presumably activated synapses.^26^

To examine the effects of ExSYTE on excitatory synaptic transmission, we generated an adeno-associated virus (AAV) expressing ExSYTE fused with a P2A cleavage site, EGFP, and DTE under a *synapsin* promoter (AAV-ExSYTE) (Figure 3H). To test various constructs, we used primary cultured neurons due to the higher throughput compared with more intact systems. We infected primary hippocampal neurons with AAV-ExSYTE (Figure 3H) and measured miniature excitatory postsynaptic synaptic currents (mEPSCs) in whole-cell recording configuration (Vh = −70 mV) (Figures S3E–S3H). Dox mediated a strong reduction in mEPSC frequency and a modest reduction in mEPSC amplitude both in naive neurons and H64A-expressing neurons in culture (Figures S3E and S3F). This observation is not entirely surprising given the “off-target” effects of this tetracycline.^29^ In contrast, in ExSYTE-expressing neurons, Dox modestly but significantly increased mEPSC amplitude and had no effect on mEPSC frequency (Figure S3G). Remarkably, Dox significantly increased both mEPSC amplitude and frequency in ExSYTE-expressing neurons when comparing H64A and naive neurons. As an alternative approach, we examined the synaptic localization of AMPARs using super-resolution stimulated emission depletion (STED) microscopy (Figures 3I and 3J) and immunostaining with anti-GluA1 and anti-PSD-95 antibodies. In the absence of Dox treatment, only 29.3% ± 4.6% and 29.1% ± 3.7% of GluA1 colocalizes with PSD-95 in neurons expressing ExSYTE or ExSYTE^H64A^, respectively. Upon Dox treatment (10 μM for 20 min), GluA1 colocalization with PSD-95 increased significantly to 49.0% ± 4.0% in neurons expressing ExSYTE, but not ExSYTE^H64A^ (28.7% ± 3.2%) (Figure 3J). Altogether, these results suggest that Dox-mediated activation of ExSYTE induced AMPAR localization at synapses in ExSYTE-expressing primary cultured neurons.

### Subthreshold training induces *c-Fos* promoter-driven ExSYTE expression, but not freezing

**Figure 4.**
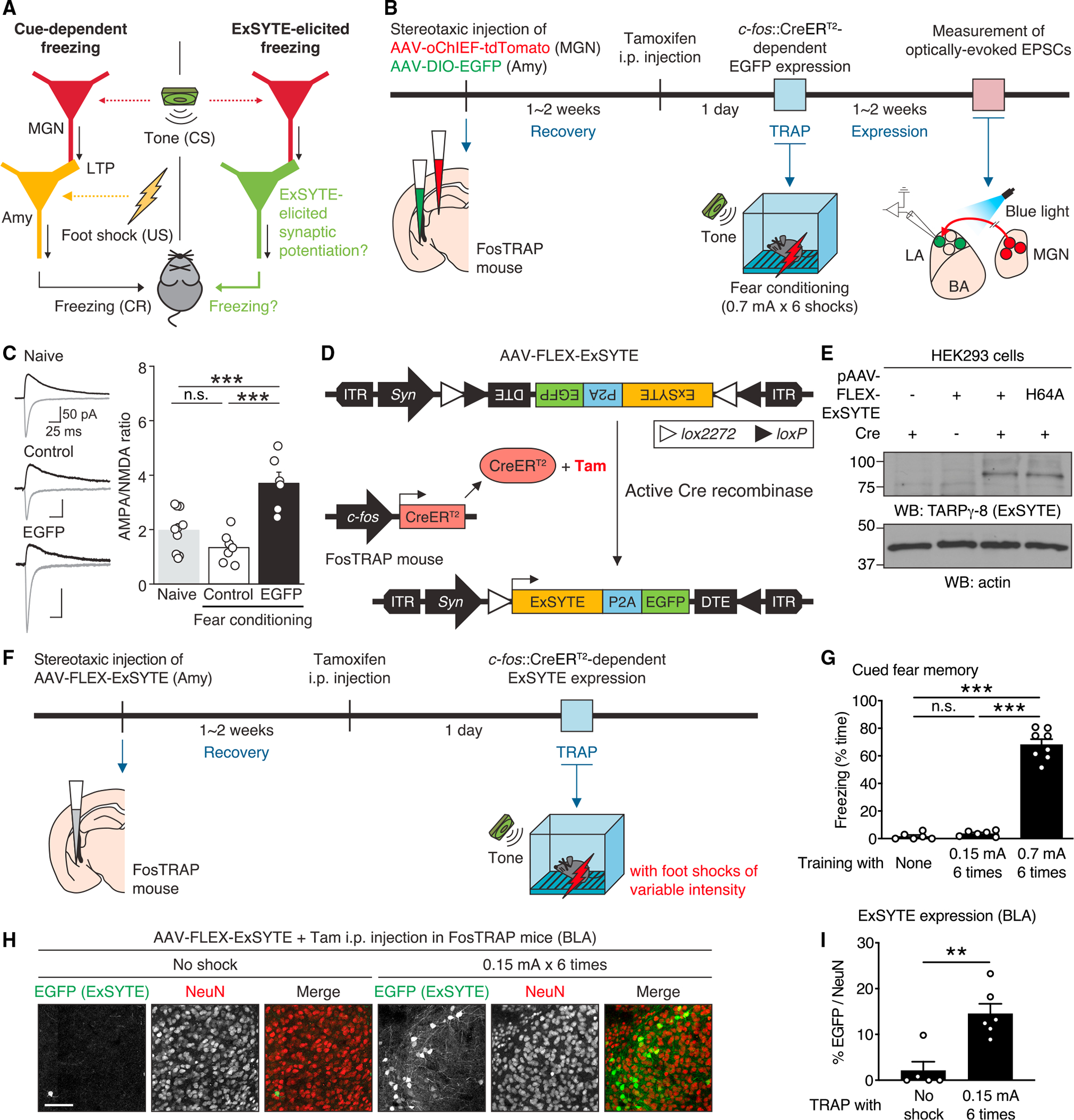
Subthreshold FC induces *c-Fos*-dependent ExSYTE expression, but not fear memory (A) Working model illustrating cue-dependent versus ExSYTE-elicited freezing. A tone (CS) coupled with a foot shock (US) induces mouse freezing (CR), as well asLTP at medial geniculate nucleus (MGN)-amygdala (Amy) synapses (left). ExSYTE expression in the amygdala with Dox administration is expected to mimic LTP and elicit freezing. (B) Schematic diagram showing that FosTRAP mice injected with AAV-oChIEF-tdTomato in the MGN and AAV-DIO-EGFP in the amygdala were trained with a conventional US condition (0.7 mA, six times) 24 h after an intraperitoneal injection of tamoxifen (Tam; 150 mg/kg body weight). (C) AMPAR- and NMDAR-EPSCs evoked by light stimulation of MGN axons were measured in EGFP-positive (EGFP) or -negative (Control) cells of acute brain slices as well as naive cells from untreated WT mice. AMPA/NMDA ratio was increased in EGFP cells (n = 7 cells from three animals) as compared with control (n = 6 cells from three animals) and naive WT cells (n = 10 cells from five animals). Representative averaged EPSC traces (left) and summary plot (right). (D–F) Spatiotemporal control of ExSYTE expression using FosTRAP system. Transcription of ExSYTE-P2A-EGFP with DTE in AAV-FLEX-ExSYTE is initiated after Cre recombinase-dependent inversion by the FLEX switch (D). Protein expression monitored by western blots with anti-TARPγ–8 antibody (E) showed that ExSYTE and ExSYTE^H64A^ as well as Actin as control were expressed at comparable levels in anti-TARPγ–8 western blots of HEK cells transfected with FLEX-ExSYTE or FLEX-ExSYTE^H64A^ in a Cre recombinase-dependent manner. (F) The AAV-FLEX-ExSYTE was injected into the amygdala of FosTRAP mice. After 1–2 weeks of recovery, Tam (150 mg/kg body weight) was injected intraperitoneally, and mice were subjected to cued FC with various foot body weight shocks 24 h later. (G) Naive mice were trained with tones (2.8 kHz, 90 dB) and different paradigms of foot shocks as indicated, and cued fear memory was examined the next day. Mice conditioned with six tone-mild (0.15 mA) foot shock pairings did not freeze, similar to non-conditioned mice (None). Mice conditioned with six tone-conventional (0.7 mA) foot shock pairing showed substantial freezing (n = 8 animals). (H and I) ExSYTE expression in mice conditioned with mild foot shocks. Mice were injected with AAV-FLEX-ExSYTE as in (F). Images were taken as tiled images. Approximately 15% of NeuN^+^ neurons were GFP^+^ in the BLA (n = 6 animals). Scale bar, 100 μm. Data are presented as mean ± SEM. n.s., non-significant, *p < 0.05, **p < 0.01, ***p < 0.001, one-way ANOVA followed by post hoc Tukey’s multiple comparisons (C), Kruskal-Wallis test followed by Dunn’s multiple comparisons test (G) and unpaired Student’s t test (I).

Based on prior characterizations of synaptic plasticity interventions in the amygdala and the neural circuitry for cued FC,^13,30^ we examined whether ExSYTE can mimic synaptically induced LTP and elicit freezing (Figure 4A). FC activates the *c-Fos* promoter in a limited number of neurons, and optogenetic activation of these neurons evokes freezing, suggesting that c-Fos-positive neurons upon FC may form memory engram.^16,18,31^ Thus, we first examined changes in synaptic transmission in c-Fos-positive neurons of acute amygdala slices following FC with strong unconditioned stimulus (US, 0.7-mA shock, six times) by specifically analyzing synaptic inputs from the medial geniculate nucleus (MGN), which conveys the conditioned stimulus (CS) to the lateral amygdala (LA).^13^ To identify c-Fos-positive neurons upon FC, we used a *c-Fos* promoter-based TRAP (targeted recombination in active populations [FosTRAP]) system that enables the expression of a protein in neurons upon training induced *c-Fos* activation.^32^ We stereotaxically injected FosTRAP mice with AAV carrying oChIEF-tdTomato^33^ and Cre-dependent DIO-EGFP into the MGN and the amygdala, respectively (Figures 4B and S4A). After 1–2 weeks of recovery, the mice were subjected to cued FC training to promote the expression of tamoxifen (Tam)-inducible Cre recombinase (CreER^T2^) under the *c-Fos* promoter presumably in neurons activated strongly during cued FC (Figure 4B). During the training period, sufficient Tam concentration for CreER^T2^ activation was provided in the brain by intraperitoneal injection of Tam 1 day before training.^32,34^ This TRAP system combined with AAV-DIO-EGFP allows EGFP to be expressed in neurons activated during cued FC. We prepared acute brain slices 1–2 weeks after TRAP and recorded from LA neurons AMPAR- and NMDAR-mediated excitatory postsynaptic currents (EPSCs) evoked by light stimulation of MGN axons. The AMPA/NMDA ratio at MGN-LA synapses was significantly increased in EGFP-positive cells, but not in EGFP-negative control cells in the same slice, nor in LA cells of untreated WT mice (Naive) (Figure 4C; Naive: 1.97 ± 0.2, n = 10 cells from five animals; Control: 1.34 ± 0.2, n = 7 cells from three animals; EGFP: 3.70 ± 0.4, n = 6 cells from three animals; Naive versus EGFP: p < 0.001, Control versus EGFP: p < 0.001, Naive versus Control: p = 0.25, one-way ANOVA followed by post hoc Tukey’s multiple comparisons). These results indicate that only c-Fos-positive LA cells activated during cued FC undergo a potentiation in AMPAR-mediated synaptic transmission consistent with previous observations.^13,35^

To selectively express ExSYTE in the c-Fos-positive neurons, we used the FLEX system.^36^ We generated an AAV carrying a *synapsin* promoter-driven FLEX-ExSYTE (AAV-FLEX-ExSYTE) or inactive ExSYTE^H64A^ with P2A-EGFP-DTE (Figure 4D). Cre-dependent expression of ExSYTE and ExSYTE^H64A^ (molecular weight ≈83 kDa each) was confirmed at similar levels in transfected HEK293 cells (Figure 4E). This AAV was stereotaxically injected into the amygdala of FosTRAP mice, followed by a similar procedure to Figure 4B but utilizing foot shocks (US) of variable intensity (Figure 4F).

We established a subthreshold training condition sufficient for *c-Fos*-dependent expression of ExSYTE, but not for evoking freezing in mice. We delivered aversive foot shocks (US; 2 s) of variable intensity at the end of each tone (CS; 2.8 kHz, 90 dB, 20 s). When trained with a strong shock (0.7 mA for 2 s, six times), WT mice showed 68.3% ± 3.8% time freezing (defined as 0.5 s without moving) upon tone stimulation (Figure 4G). However, WT mice did not freeze when a weaker shock (0.15 mA for 2 s, six times) or no shock was used (Figure 4G; 3.7% ± 0.8% and 1.7% ± 1.0%, respectively). Comparing ExSYTE expression in FosTRAP mice under various US conditions, we found that EGFP was barely detectable among many NeuN-positive neurons in the no shock condition (Figures 4H, 4I, S4B, and S4C). In contrast, both the subthreshold US (0.15-mA shocks, six times) and strong US (0.7-mA shocks, six times) increased the numbers of EGFP-positive neurons significantly in basolateral amygdala (BLA) and CeA (Figures 4H, 4I, S4B, and S4C). Compared with the *synapsin* promoter-driven (pan-neuronal) expression of ExSYTE (Figures S4D and S4E; 61.3% ± 7.9% and 38.5% ± 3.9% of neurons in BLA and CeA, respectively), this *c-Fos* promoter-driven expression of ExSYTE (FLEX-ExSYTE) was expressed in a lower number of amygdala neurons (Figures S4B and S4C; 14.5% ± 2.1% and 17.5% ± 2.1% of neurons in BLA and CeA, respectively). Altogether, these results indicate that subthreshold cued FC, which does not evoke freezing, induces neuron-type specific ExSYTE expression.

### Active ExSYTE mimics LTP

**Figure 5.**
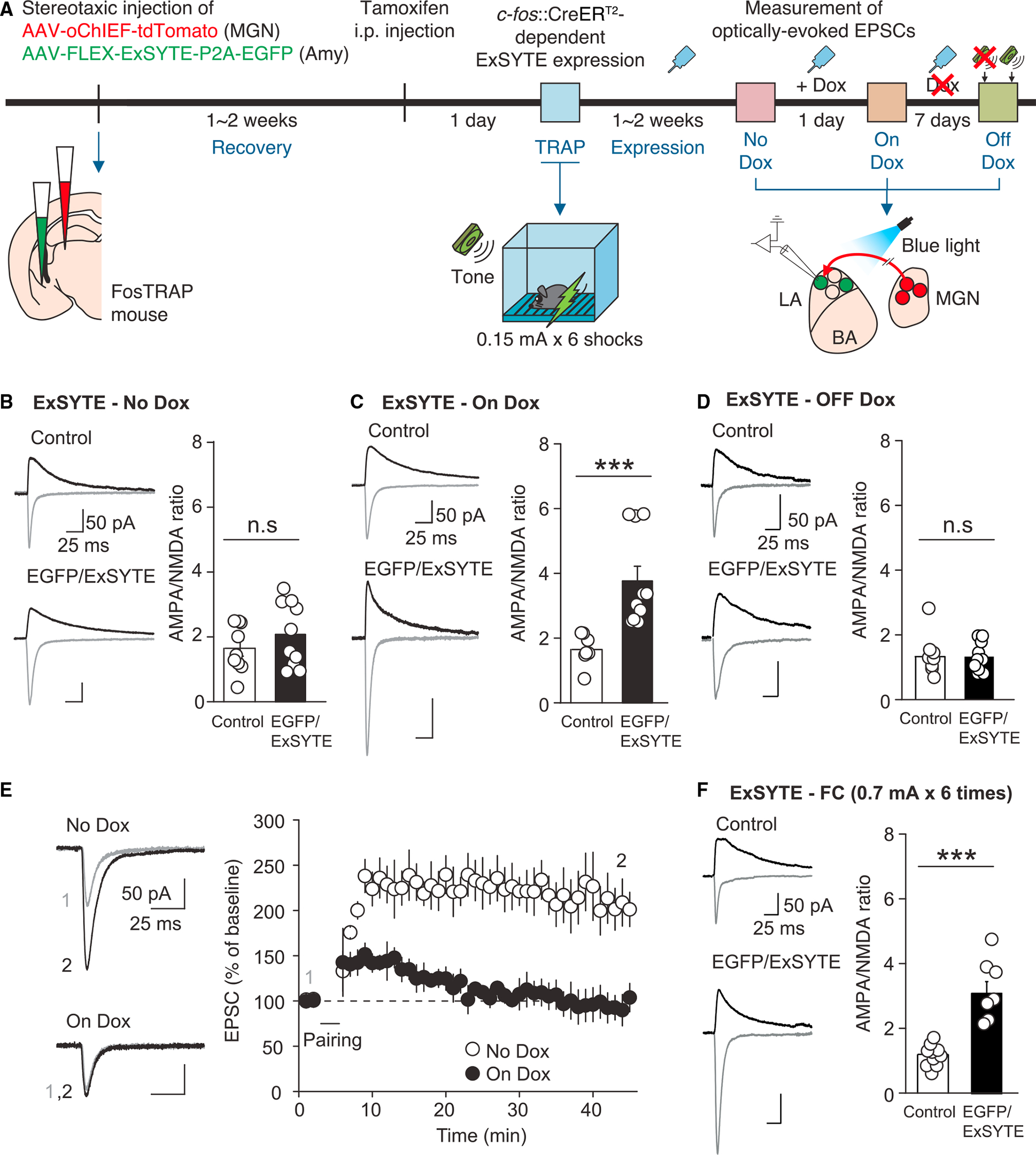
Active ExSYTE expression increases synaptic AMPAR-mediated transmission and occludes LTP (A) Schematic diagram showing that FosTRAP mice injected with AAV-oChIEF-tdTomato in the MGN and AAV-FLEX-ExSYTE.P2A.EGFP in the amygdala were-trained with a subthreshold US condition (0.15 mA, six times) 24 h after an intraperitoneal injection of tamoxifen (Tam; 150 mg/kg body weight). Optically evoked AMPAR- and NMDAR-EPSCs at the MGN-LA synapses were measured in EGFP-positive (EGFP/ExSYTE) or EGFP-negative (control) cells of acute brain slices before (No Dox) or after 1 day Dox administration (On Dox) or 7 days after Dox removal (Off Dox). (B) AMPA/NMDA ratio does not differ between control (n = 9 cells from three animals) and EGFP/ExSYTE cells (n = 10 cells from four animals), before Dox administration. (C) After 1 day Dox administration, AMPA/NMDA ratio increased in EGFP/ExSYTE cells (n = 10 cells from four animals) as compared with control cells (n = 7 cells from four animals). (D) Seven days after Dox removal, AMPA/NMDA ratio in EGFP/ExSYTE cells (n = 10 cells from six animals) was similar to controls (n = 9 cells from six animals). (E) LTP was abolished in EGFP/ExSYTE cells after Dox administration (n = 5 cells from four animals) as compared with No Dox (n = 5 cells from three animals). Representative averaged EPSCs (left) and summary plot (right). (F) Following the experimental timeline shown in (A), mice were trained with a conventional US condition (0.7 mA, six times) and 1–2 weeks later optically evoked AMPA/NMDA ratio was measured. AMPA/NMDA ratio was increased in EGFP/ExSYTE cells (n = 8 cells from seven mice) as compared with controls (n = 12 cells from seven mice), suggesting that ExSYTE expression does not affect FC-induced AMPAR potentiation. Data are presented as mean ± SEM. n.s., non-significant, ***p < 0.001, paired Student’s t test (B and D) and Mann-Whitney U test (C and F).

To examine whether ExSYTE mimics synaptic potentiation observed in mice undergoing cued FC, we injected FosTRAP mice with AAV-oChIEF-tdTomato into the MGN for specific optical stimulation of MGN axons, and AAV-FLEX-ExSYTE into the amygdala (Figure 5A), followed by subthreshold cued FC to express ExSYTE in c-Fos-positive neurons in a Cre recombinase-dependent manner. One to 2 weeks after FosTRAP induction with a subthreshold US, we prepared acute brain slices before administration of Dox (No Dox) and measured optically evoked AMPAR- and NMDAR-mediated EPSCs from EGFP/ExSYTE-positive and negative neurons (Figure 5A). Unlike EGFP/ExSYTE-positive neurons upon activation of the *c-Fos* promoter by strong US (Figure 4C), the AMPA/NMDA ratio in EGFP-positive neurons before Dox was not affected by the subthreshold FC, similar to EGFP-negative control neurons, indicating that ExSYTE expression does not alter basal transmission at MGN-LA synapses (Figure 5B; Control cells: 1.65 ± 0.2, n = 9 cells from three animals; EGFP/ExSYTE cells: 2.07 ± 0.3, n = 10 cells from four animals; p = 0.31, unpaired Student’s t test). These results suggest that the US intensity determines the magnitude of synaptic potentiation in *c-Fos*-activated neurons, and our subthreshold training does not affect synaptic strength.

In contrast, the AMPA/NMDA ratio at MGN-LA synapses was substantially increased in EGFP/ExSYTE cells as compared with control cells when brain slices were prepared 1 day after Dox application via drinking water (On Dox) (Figure 5C; Control cells: 1.65 ± 0.2, n = 7 cells from four animals; EGFP/ExSYTE cells: 3.76 ± 0.4, n = 10 cells from four animals; p < 0.001, Mann-Whitney U test), supporting the synaptic insertion of AMPARs in Dox-activated ExSYTE neurons. Furthermore, 7 days after Dox removal in drinking water, the AMPA/NMDA ratio was indistinguishable between EGFP/ExSYTE cells and control cells (Figure 5D, Control cells: 1.37 ± 0.2, n = 9 cells from six animals; EGFP/ExSYTE cells: 1.35 ± 0.1, n = 10 cells from six animals; p = 1, Mann-Whitney U test), suggesting that the synaptic insertion of AMPAR is reversible upon Dox removal. Last, injecting mice with inactive ExSYTE^H64A^ (same experimental timeline as in Figure 5A) had no effect on the AMPA/NMDA ratio (Figures S5A and S5B, No Dox: Control cells: 1.49 ± 0.2, n = 7 cells from four animals; EGFP/ExSYTE^H64A^ cells: 1.26 ± 0.1, n = 7 cells from four animals; p = 0.2799, unpaired Student’s t test; On Dox: Control cells: 1.21 ± 0.1, n = 9 cells from six animals; EGFP/ExSYTE^H64A^ cells: 1.4 ± 0.2, n = 9 cells from six animals; p = 0.3536, Mann-Whitney U test). Together, these results strongly suggest that AMPAR-mediated synaptic transmission is potentiated upon Dox treatment in ExSYTE-expressing neurons.

To test whether LTP and ExSYTE-mediated potentiation share a common mechanism, we examined MGN-LA LTP in ExSYTE or ExSYTE^H64A^-expressing cells before and 1 day after Dox. In Dox-treated animal experiments, Dox was also added to brain slices both during slice preparation and recordings. A pairing protocol consisting of light stimulation at 3 Hz for 3 min paired with postsynaptic depolarization to 0 mV,^35^ triggered robust LTP of MGN-LA EPSCs in ExSYTE-expressing cells in the absence of Dox (Figure 5E; No Dox: 205% ± 17%, n = 5 cells from three animals; p < 0.01, paired Student’s t test) as well as in ExSYTE^H64A^-expresssing cells in the presence and absence of Dox (Figure S5C; No Dox: 227% ± 7.4%, n = 5 cells from four animals; On Dox: 237% ± 7.3%, n = 4 cells from three animals). In contrast, no MGN-LA LTP was induced 1 day after Dox application (Figure 5E; On Dox: 97% ± 16%, n = 5 cells from four animals; p = 0.85, paired Student’s t test; No Dox versus On Dox: p < 0.01, unpaired Student’s t test), suggesting that MGN-LA LTP was occluded. Alternatively, ExSYTE expression by itself could have impaired LTP. To address this possibility, we examined FC-induced AMPAR potentiation in neurons expressing ExSYTE (as in Figure 4C). We found that FC increased the AMPA/NMDA ratio in ExSYTE-expressing cells (Figure 5F, Control cells: 1.2 ± 0.1, n = 12 cells from seven animals; EGFP/ExSYTE cells: 3.08 ± 0.3, n = 8 cells from seven animals; p < 0.001, unpaired Student’s t test) similar to that in EGFP-expressing cells (Figure 4C), indicating that ExSYTE expression does not affect the ability of MGN-LA synapses to undergo LTP. We therefore conclude that ExSYTE-mediated potentiation and synaptically induced LTP share a common mechanism.

### Active ExSYTE expression in subthreshold training-induced c-Fos-positive neurons evokes FC-like freezing

**Figure 6.**
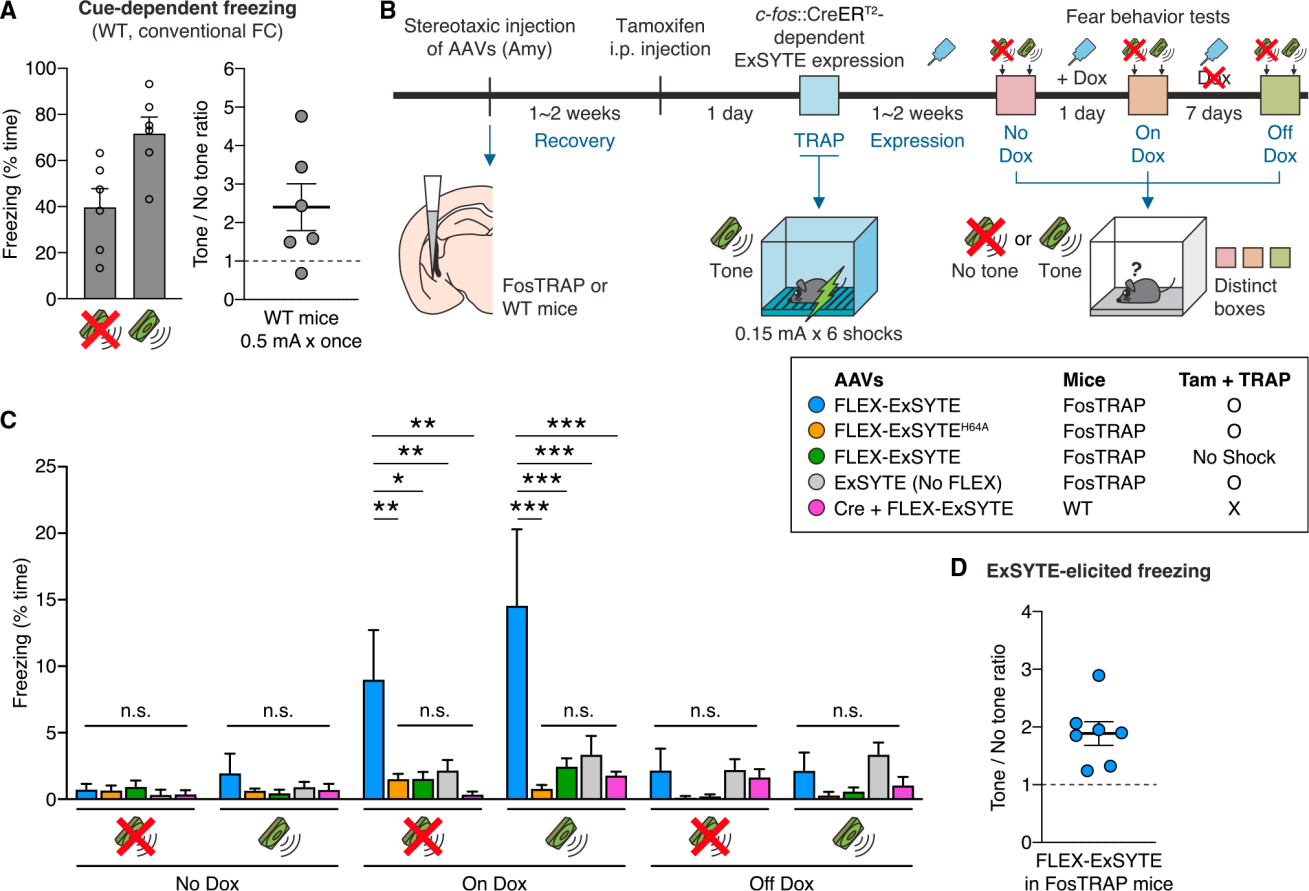
ExSYTE activation in a subset of amygdala neurons elicits freezing (A) The freezing rate and ratio of conventionally fear-conditioned WT mouse data (0.5 mA shock, once) were plotted (n = 6 animals). (B) FosTRAP mice injected with either AAV-FLEX-ExSYTE, AAV-FLEX-ExSYTE^H64A^, or AAV-ExSYTE (non-FLEX) were trained with or without a subthreshold US condition (0.15 mA, six times) 24 h after an intraperitoneal injection of tamoxifen (Tam; 150 mg/kg body weight). Freezing was monitored with or without a tone (2.8 kHz, 90 dB) before Dox (No Dox), 1 day after Dox administration (On Dox), and 7 days after Dox withdrawal (Off Dox) in different boxes. As a control group, WT mice were co-injected with a mix of AAV-Cre and AAV-FLEX-ExSYTE, and subjected to the freezing tests. (C and D) FosTRAP mice injected with AAV-FLEX-ExSYTE and subjected to the subthreshold US conditioning (blue) showed freezing upon Dox administration in drinking water (2 mg/mL) without or with tone. The freezing ratio of each mouse with or without a tone increased consistently (D, n = 6 animals). No freezing behavior was observed in FosTRAP mice injected with AAV-FLEX-ExSYTE^H64A^ and subjected to the subthreshold US conditioning (orange), in mice injected with AAV-FLEX-ExSYTE without foot shocks (green), or in mice injected with AAV-ExSYTE and subjected to foot shocks (gray) (C). WT mice co-injected with a mix of AAV-Cre and AAV-FLEX-ExSYTE (purple) showed no significant freezing despite a similar number of ExSYTE-expressing neurons (C). n = 7 for “FLEX-ExSYTE in FosTRAP + TRAP,” n = 6 for “FLEX-ExSYTE^H64A^ in FosTRAP + TRAP,” n = 5 for “FLEX-ExSYTE in FosTRAP + No shock,” n = 9 for “ExSYTE in FosTRAP + TRAP,” and n = 5 for “Cre + FLEX-ExSYTE in WT.” Data are presented as mean ± SEM. n.s., non-significant, *p < 0.05, **p < 0.01, ***p < 0.001, two-way ANOVA followed by post hoc Tukey’s multiple comparisons (C).

We next tested whether ExSYTE-dependent synaptic AMPAR potentiation can elicit FC-like freezing. It is known that conventional FC generates both non-associative (pre-CS) and tone-dependent (CS) freezing proportionally,^37,38^ which we first confirmed in mice subjected to cued FC with strong US (0.5-mA shock, once) (Figure 6A). Remarkably, analyzing the CS/pre-CS freezing ratio (i.e., Tone/No tone ratio) provides a more accurate measurement of the tone-dependent memory, and we confirmed the increase of the CS/pre-CS freezing ratio (Figure 6A). Thus, we hypothesized that the CS/pre-CS freezing ratio with pre-CS and CS freezing is increased in active ExSYTE-expressing mice.

We examined FC-like freezing in mice expressing ExSYTE in c-Fos-positive amygdala neurons upon Dox application (Figure 6B). To this end, we stereotaxically injected AAV-FLEX-ExSYTE into the amygdala of WT or FosTRAP mice and allowed them to recover for 1 to 2 weeks before FosTRAP induction. One to 2 weeks after FosTRAP induction with a subthreshold US, we examined fear behavior in TRAP mice with Dox activation of ExSYTE, a manipulation that potentiates AMPAR-mediated transmission in a Dox-dependent manner (Figures 5C and 5D). We examined Dox-dependent responses with or without tone stimulation. To minimize the contribution of contextual information to fear behavior, we performed each test in a different box (illustrated as different colors of boxes in Figure 6B).

The TRAP mice with intra-amygdala injections of AAV-FLEX-ExSYTE did not freeze before Dox application (No Dox) (Figure 6C). In contrast, 1 day after Dox exposure via drinking water (On Dox), the mice displayed freezing with or without tone stimulation, i.e., associative learning or generalization, respectively (Figure 6C). Relative to their behavior without tone exposure, each FosTRAP mouse consistently showed an increase in freezing upon tone stimulation (Figure 6D). This increase in the ratio of freezing before and during a tone (Figure 6D) was not significantly different from the ratio of freezing in WT mice subjected to strong (conventional) FC (Figure 6A). The fact that active ExSYTE and conventional FC showed comparable CS/pre-CS freezing ratios suggests that AMPAR-mediated potentiation mediated by controlling the LTP machinery is sufficient for FC-dependent freezing. Consistent with reversibility of the DR domain interaction with plasma membranes upon Dox removal (Figure 2E), ExSYTE-expressing mice did not freeze 7 days after Dox withdrawal (Off Dox) (Figure 6C), supporting the reversible effect and the specific contribution of ExSYTE to freezing.

Of note, both FosTRAP mice injected with inactive FLEX-ExSYTE^H64A^ and subjected to a subthreshold US or injected with active FLEX-ExSYTE and never exposed to the subthreshold US (i.e., no shock), and FosTRAP mice injected with (non-FLEX) ExSYTE and subjected to a subthreshold US, did not showed any significant freezing before Dox application (No Dox), 1 day after Dox application (On Dox), or 7 days after Dox removal (Off Dox) (Figures 6B and 6C). Despite an increase in freezing from FLEX-ExSYTE mice, overall locomotion was not significantly altered compared with control groups (Figure S6A). Pan-neuronal expression of ExSYTE in WT mice also showed no significant increase in freezing (Figure S6B). Further, we also confirmed the increase in the AMPA/NMDA ratio at MGN-LA synapses in Dox-treated neurons expressing FLEX-ExSYTE and *EF1α* promoter-Cre recombinase,^39^ as compared with control cells (Figure S6C; Control cells: 1.26 ± 0.16, n = 8 cells from five animals; EGFP/ExSYTE cells: 3.52 ± 0.3, n = 8 cells from five animals; p < 0.001, unpaired t test). After the completion of all behavioral testing, ExSYTE expression (as reflected by EGFP signal) in the BLA was confirmed in all US-exposed mice (Figures S6D–S6G), suggesting that the lack of freezing after Dox removal was not due to loss of ExSYTE expression. While we observed variability in Dox-dependent freezing, we found no correlation between AAV infection levels detected as EGFP signals in the amygdala and freezing (Figure S6H). It is therefore unlikely that freezing variability could be related to viral infection levels in the BLA despite the consistent increase in cue-dependent freezing in those mice.

Lastly, the difference in freezing could be due to the *c-Fos*-dependent neuronal identity or the percentage of neurons expressing ExSYTE because behavioral changes were observed only when ExSYTE was selectively expressed in c-Fos-positive neurons (14.5% ± 2.1% of BLA and 17.5% ± 2.1% of CeA), but not when expressed non-selectively using a *synapsin* promoter (61.3% ± 7.9% of BLA and 38.5% ± 3.9% of CeA) in the amygdala (Figures 4H, 4I, S4C, and S4E). To further investigate this possibility, we co-injected AAVs carrying *EF1α* promoter-Cre recombinase^39^ and FLEX-ExSYTE with various ratios. We were able to establish a condition that expressed EGFP/ExSYTE in 14.0% ± 0.9% of BLA and 9.4% ± 0.6% of CeA neurons (Figures S6I and S6J), similar to the c-Fos-positive population of neurons (Figures 4I, S4B, and S4C). However, no freezing was observed in this condition (Cre + FLEX-ExSYTE) with or without Dox (Figure 6C). Considering the Dox-dependent increase in the AMPA/NMDA ratio in ExSYTE-expressing neurons (Figure S6C), these results indicate that the neuronal identity (c-Fos-positive), rather than the number of neurons, underlies the behavioral changes. Taken together, these results suggest that ExSYTE-mediated synaptic potentiation, specifically in amygdala neurons activated by the subthreshold US, can produce freezing behavior.

## DISCUSSION

In this study, we generated a chemogenetic tool, ExSYTE, which controls the interaction of the TARP cytoplasmic domain with the plasma membrane, and showed that this interaction is sufficient for potentiating AMPAR synaptic transmission and inducing behavioral changes. Upon chemogenetic activation with Dox, ExSYTE rapidly potentiates AMPAR-mediated transmission even in the absence of presynaptic activity. This tool allowed us to reveal a specific function of AMPAR potentiation in regulating behavior. Using a subthreshold FC protocol that induces *c-Fos* promoter activation without fear memory, we were able to express ExSYTE in subthreshold FC-activated amygdala neurons. Combining ExSYTE and this subthreshold FC protocol, we found that Dox activation of ExSYTE elicited freezing, indicating that ExSYTE-mediated potentiation of MGN-LA synapses was sufficient to generate FC-like behavior.

### ExSYTE mimics TARP-dependent LTP

We found that controlling the membrane interaction of the TARP cytoplasmic domain using ExSYTE was sufficient to strengthen AMPAR-mediated transmission and mimic LTP (Figures 3 and 5). The TARP-modified ExSYTE is a molecular tool that potentiates AMPAR-mediated transmission utilizing a critical component of the endogenous LTP machinery. ExSYTE activation by Dox increased the AMPA/NMDA ratio and occluded LTP (Figure 5), indicating that ExSYTE activation, by dissociating the TARPγ–8 cytoplasmic domain from the plasma membrane (Figures 1 and 2), is an effective way of driving AMPARs into synapses similar to LTP. ExSYTE was constructed by modifying the cytoplasmic domain of TARPs that modulates AMPAR trafficking.^23^ In the hippocampus, most AMPARs form complexes with TARPγ–8.^40^ Local injection of an antibody against the extracellular domain of the AMPAR GluA2 subunit, which inhibits lateral diffusion of AMPARs, blocks LTP and contextual learning in mice,^41^ suggesting that TARP-AMPAR complexes are inserted into synapses through lateral diffusion.

Upon LTP induction, AMPAR density is increased at the active synapse presumably through AMPAR insertion into synapses and captured by a putative slot that captures AMPARs, such as PSD-95.^6,42^ Consistent with this model, overexpression of GluA1-TARPγ–8 tandem protein restores LTP in AMPAR null neurons (GluA1/2/3 triple knockout neurons), but overexpression of GluA1-TARPγ–8Δ4 lacking its PDZ binding domain did not.^43^ Furthermore, RNAi-based triple knockdown of PSD-93/95/SAP102 showed no LTP.^44^ Notably, in both cases, a substantial reduction in basal transmission was observed, comparable to the one observed in SAP102 knockdown neurons from PSD-93/95 double knockout mice.^45^ In contrast, PSD-95 knockout mice showed enhanced LTP,^46,47^ and TARPγ–8Δ4 knockin mice, which cannot interact with PSD-95, showed unaltered LTP.^27^ These results indicate that PSD-95 is not required for LTP of AMPAR-mediated synaptic transmission. This apparent discrepancy could be due to the distinct experimental systems, gene-targeting mice, and the tandem protein-overexpression system that was utilized.

A reduction in basal synaptic transmission by deleting PSD-95 and the TARPγ–8 PDZ binding motif was observed consistently by several groups,^27,43–46,48^ supporting essential roles of PSD-95 and the TARPγ–8 PDZ binding motif in basal transmission. Recently, the second non-C-terminal PDZ binding site was proposed in TARPγ–8, and the mutation in this domain altered basal synaptic transmission by re-introducing GluA1-TARPγ–8 tandem protein into AMPAR null neurons.^49^ Conversely, the same system with different GluA1-TARPγ–8 tandem mutant proteins showed that the C-terminal PDZ binding domain of TARPγ–8 is sufficient for enhancing AMPAR transmission for LTP.^50^ The roles of the second non-C-terminal PDZ binding site should be tested by utilizing gene-targeting mice that presumably maintain protein expressions at endogenous levels.

TARPγ–8 phosphorylation enhances AMPAR transmission and is required for LTP.^9^ Despite the unresolved roles of PSD-95 in LTP, a putative slot protein like PSD-95 localizes at synapses but does not have access to extrasynaptic AMPARs. Therefore, there must be a step that increases AMPAR density at synapses before these receptors are captured by PSD-95. Here we found that the dissociation of the TARPγ–8 cytoplasmic domain from the plasma membrane is sufficient to induce AMPAR potentiation without neuronal stimulation. Our finding raises the possibility that the release of the TARP/AMPAR complex from extrasynaptic sites is a step required for potentiating synaptic transmission before TARP interaction with PSD-95. Furthermore, we showed that the Dox withdrawal reverses the Dox-potentiated AMPAR transmission to the basal level in ExSYTE-expressing neurons (Figure 5D). Perhaps, the lack of the PDZ binding motif in ExSYTE enables reversibility of ExSYTE in AMPAR potentiation and behaviors (Figures 5D and 6C), and TARP-PSD-95 interaction may play roles in this later step after TARPγ–8 insertion into synapses. Future experiments that visualize each step in real time and at high anatomical resolution upon LTP induction are warranted.

### Achemogenetictooltocontrollipid-proteininteractions

A key feature of ExSYTE is its lipid-interacting module that can chemogenetically dissociate from lipids. Lipid-protein interactions are largely classified into two types: single lipid-protein interactions and positive-charged proteins interacting electrostatically with negatively charged membranes. Previous studies have reported chemogenetic and optogenetic approaches to control single lipid-protein interactions.^51,52^ However, ExSYTE is a chemogenetic tool that controls the electrostatic interaction of a protein with membranes due to the integration of a DR module. Notably, this domain could be broadly applied to control other protein-membrane interactions.

### Active ExSYTE induces a conditioned response

We found that Dox/ExSYTE-mediated synaptic potentiation evokes freezing in mice specifically expressing ExSYTE in activated amygdala neurons stimulated by subthreshold FC. Because Dox-activated ExSYTE promotes postsynaptic insertion of AMPARs, these findings suggest that postsynaptic potentiation in the amygdala is sufficient to evoke the conditioned response (CR), consistent with the notion that AMPAR-LTP can support associative learning and memory.

The relationship between synaptic plasticity in the amygdala and FC was inferred early from correlative evidence.^4,53,54^ Impairment of behavioral memory in mice lacking synaptic plasticity due to interventions that target critical genes encoding plasticity-associated proteins such as GluA1,^55,56^ CaMKII,^57,58^ and TARPγ–8,^9^ or that manipulate LTP and LTD,^13^ provided support for this inference. However, due to temporal limitations of mouse gene targeting, limited cellular resolution in LTP and LTD as compared with the FosTRAP system,^32^ and the non-synaptic changes (e.g., excitability) that can be associated with LTP induction protocols,^14,15^ it has not yet been possible to demonstrate that postsynaptic potentiation alone is sufficient for fear learning. Our chemogenetic approach directly addresses this long-standing question.

Dox-induced freezing in our ExSYTE-expressing mice (~15%) was close to that seen with optogenetic and chemogenetic reactivation of cells activated during prior FC (20%–30%),^16,31^ indicating that postsynaptic potentiation modulated by ExSYTE significantly contributes to freezing behavior. These non-behavioral methods produce less freezing than FC with a US (~50%).^9,58^ This difference could be due to the selective expression of ExSYTE in amygdala or the number of neurons activated by a conventional versus a subthreshold US. In our case, AAV-based ExSYTE expression was limited to a local region in the amygdala and the subthreshold US activated a smaller number of c-Fos-positive neurons compared with the conventional US (Figures 4I and S4C). Mice expressing ExSYTE in subthreshold FC-activated neurons in the whole brain might show more robust behavioral outcomes. Physiological changes within the amygdala may be insufficient to create full-strength cued FC, supporting the notion that other brain regions and/or mechanisms are likely involved.^59–61^ We also noted that freezing was not long-lasting upon Dox removal, which is likely due to the reversible property of the DR module in ExSYTE (Figure 2).

### Versatile application of ExSYTE and FosTRAP system to other behaviors

The tightly controlled ExSYTE approach can be applied to any brain region accessible to AAV stereotaxic injection, such as the hippocampus, a brain region that is critical for contextual, but not cued, FC.^16,18,61^ It is worth noting that “our subthreshold training protocol” induced c-Fos expression without developing any freezing (Figure 4G) or AMPAR potentiation (Figures 4C and 5B). Although both training protocols activate the *c-Fos* promoter (Figures S4B and S4C), AMPAR-mediated transmission was increased only in c-Fos-positive neurons under the conventional FC training (Figure 4C), but not in those under subthreshold training (Figure 5B). These findings indicate that the conventional FC induced AMPAR-mediated transmission in c-Fos-positive neurons (consistent with the role of LTP in FC), whereas our subthreshold training induced *c-Fos* promoter activation without altering AMPAR-mediated transmission. These results indicate that LTP was not induced by the subthreshold protocol. This unexpected dissociation between *c-Fos* promoter activity and synaptic changes suggests that c-Fos expression is more sensitive to neuronal activity than LTP.

### Limitations of the study

In this study, we have generated a chemogenetic tool that can mimic LTP of AMPAR-mediated transmission and FC. While activation of ExSYTE was sufficient to enhance AMPAR insertion in WT mice, targeting TRAPed neurons (i.e., Fos-positive neurons) was required to elicit a freezing behavior. To express ExSYTE in neurons activated during FC, we established a subthreshold FC training protocol sufficient for activating *c-Fos* promoter but not for evoking freezing in mice. Though this protocol did not trigger tone-associated freezing, it could have induced molecular changes, which, together with ExSYTE-induced LTP, promoted tone-dependent freezing. These observations suggest that memory formation may require coordinated changes in synaptic strength at the pre- and postsynaptic levels. In addition, activation of ExSYTE increased the pool of AMPARs at the synapse upon Dox application. However, the ExSYTE-mediated potentiation of AMPAR transmission was reversible after the Dox withdrawal. This observation raises the possibility that the lack of PDZ binding motif in ExSYTE enables the reversibility of both ExSYTE-induced LTP and behavior. TARP-PSD-95 interaction may play roles in a later step. Future experiments are warranted to visualize these steps in real time and at high spatial resolution upon LTP induction.

## STAR★METHODS

### RESOURCE AVAILABILITY

#### Lead contact

Further information and requests for resources and reagents should be directed to and will be fulfilled by the lead contact, Susumu Tomita (Susumu.Tomita@yale.edu).

#### Materials availability

Any materials generated in this study are being made available. Plasmids generated in this study will be deposited to Addgene.

#### Data and code availability

- Numerical data for each figure are included with the manuscript as source data. All other data are available from the authors upon request.
- This paper does not report original code.
- Any additional information required to reanalyze the data reported in this paper is available from the lead contact upon request.

### EXPERIMENTAL MODEL AND STUDY PARTICIPANT DETAILS

#### Animals

C57BL/6J mice (Stock No: 000664) and the FosTRAP (B6.129(Cg)-*Fos*^*tm1.1(cre/ERT2)Luo*^/J) heterozygous mice (Stock No: 021882)^32^ were obtained from The Jackson Laboratory. These two mouse strains were crossed to obtain wild-type and the FosTRAP heterozygous mice. Ages of study are 2–3 months of age. Both male and female mice were used for electrophysiology and histology experiments. Only male mice were used for behavioral tests to minimize the effects of estrous cycles.

All animal handling was in accordance with protocols approved by the Institutional Animal Care and Use Committee (IACUC) of Yale University (Animal Welfare Assurance# D16–00146, Animal protocol number 11029) and the Albert Einstein College of Medicine (Animal Welfare Assurance# D16–00200, Animal protocol number 00001043). Animal care and housing were provided by the Yale Animal Resource Center (YARC), in compliance with the Guide for the Care and Use of Laboratory Animals (National Academy Press, Washington, DC, 1996). Mice were housed in ventilated cages on a 12-h light/dark cycle with access to standard laboratory chow and water *ad libitum*.

#### Cell culture and transfection

HeLa, CHO, HEK293 cells were obtained from ATCC (CCL-2, CCL-61, CRL-1573) and 293AAV cells were obtained from Cell Biolab (Cat#AAV-100). Cells were maintained in DMEM supplemented with 10% (v/v) fetal bovine serum (FBS) and were cultured in a humidified atmosphere at 37°C and 5% CO_2_. CHO cells were maintained in F-12 supplemented with 10% (v/v) FBS. Transient transfection was performed using FuGENE 6 according to the manufacturer’s instructions.

#### Primary cultured hippocampal neurons

Primary hippocampal neurons were prepared from P0 mouse pups and grown on glass coverslips pre-coated with poly-D-lysine as previously described.^62^ Briefly, hippocampi were dissected, treated with papain (20 unit/ml at 37°C for 30 min), and triturated with a fire-polished glass pipet. The dissociated cells were plated and cultured at 37°C with 5% CO_2_ in Neurobasal medium containing 5% FBS, Glutamax (Gibco), B27 supplement (Gibco), and penicillin/streptomycin. Culture media were replaced with Neurobasal medium supplemented with Glutamax (Gibco), B27 supplement (Gibco), and penicillin/streptomycin 4–6 h later. At DIV14–16, cultures were transfected with 0.5 μg of ExSYTE-EGFP-DTE (or non-DTE version of ExSYTE-EGFP) and 0.5 μg of mRFP (or iRFP) plasmids using calcium phosphate. 24 to 40 h later, fluorescence images were acquired on a laser scanning confocal microscope (LSM 710; Zeiss) or an UltraVIEW VoX spinning disk confocal microscope (Perkin Elmer) equipped with Perfect Focus, temperature-controlled stage, 14-bit EMCCD camera (Hamamatsu C9100–50) and controlled by Volocity software (Perkin Elmer).

### METHOD DETAILS

#### Antibodies

The following antibodies were used: rabbit polyclonal antibody to GluA1^27^; guinea pig polyclonal antibodies to GFP^63^ and TARPγ–8 ^27^; and mouse monoclonal antibodies to NeuN (Millipore) and actin (Millipore).

#### Constructs

The cDNA encoding a bacterial tetracycline repressor (TetR) was amplified from pCAGTetRnls (Addgene #26599).^19^ Single or double TetR fragments with or without a polybasic amphiphilic helix ‘KRϕ’^20,21^ were generated by overlap extension PCR with the primers in Table S1 and inserted into a pEGFP-C2 vector (Clontech) using XhoI and EcoRI sites. Variations of the polybasic lipid-binding domain were generated by mutagenesis PCR with the primers in Table S1. The codon-optimized version of TetR (oTetR) was acquired by PCR from pTet-Off-Advanced (Clontech #631070). The construct encoding the final Dox-Responsive module (DR) was generated by overlap extension PCR with the primers in Table S1 and insertion into a pEGFP-C2 vector using XhoI and EcoRI sites. To generate the mutant DR^H64A^ control, an alanine substitution was introduced into the histidine 64 residue of the oTetRs (H64A) by mutagenesis PCR with the primers in Table S1.

To generate ExSYTE constructs by assembling the DR module with a TARPγ–8 template, a DNA fragment encoding the DR module was ligated with two cDNA fragments corresponding to the N-terminal TARPγ–8 (1–256 amino acids) and its C-terminal tail (358–423 amino acids) using XhoI and SpeI sites into a Gateway entry vector. The pGEM-HE-ExSYTE (for expression in *Xenopus laevis* oocytes), pAAV-*Syn*-ExSYTE, and pAAV-*Syn*-FLEX-ExSYTE (for AAV production) were made by Gateway LR recombination. For a cistronic expression marker, a DNA fragment encoding P2A-EGFP^64^ was generated by overlap extension PCR with the primers in Table S1 and assembled next to the TARPγ–8 C-terminal tail using PstI and XbaI sites. For neuronal input specificity, the dendritic targeting element (DTE) of rat *Arc* mRNA (Accession # NM_019361)^24–26^ was amplified by PCR from a rat hippocampal mRNA library with the primers in Table S1 and inserted after the stop codon of EGFP in the pAAV plasmids using XbaI sites. The pGEM-HE-HA-GluA1 construct was described.^65^ To express EGFP-tagged ExSYTE (ExSYTE-EGFP) in cultured neurons, the last five amino acids of P2A (ENPGP; GAG AAC CCT GGA CCT) were removed from ExSYTE-P2A-EGFP by mutagenesis PCR with the primers (Table S1) and sub-cloned into pGW1 vector. All constructs will be deposited to Addgene.

pCAGTetRnls, piRFP, AAV-oChIEF-tdTomato, and pAAV-hSyn-DIO-EGFP were a gift from Peter Andrews (Addgene plasmid #26599; http://n2t.net/addgene:26599; RRID:Addgene_26599),^19^ Vladislav Verkhusha (Addgene plasmid #31857; http://n2t.net/addgene:31857; RRID:Addgene_31857),^28^ Roger Tsien (Addgene plasmid #50977; http://n2t.net/addgene:50977; RRID:Addgene_50977),^33^ and Bryan Roth (Addgene plasmid #50457; http://n2t.net/addgene:50457; RRID:Addgene_50457), respectively.

#### Cell imaging

Live imaging with a spinning-disc confocal microscope or a total internal reflection fluorescence (TIRF) microscope was performed as previously described.^66^ Briefly, cells were plated on 35 mm glass-bottom dishes (MatTek Corporation), and imaged at 37°C approximately 18 h after transfection. Before imaging, cells were transferred to a pre-warmed buffer containing 10 mM HEPES (pH 7.4), 137 mM NaCl, 2.5 mM KCl, 2 mM CaCl_2_, and 1.3 mM MgCl_2_. Time-lapse confocal fluorescence images were acquired on an UltraVIEW VoX spinning disk confocal microscope (Perkin Elmer) equipped with Perfect Focus, temperature-controlled stage, 14-bit EMCCD camera (Hamamatsu C9100–50) and controlled by Volocity software (Perkin Elmer). Time-lapse TIRF micrographs were acquired on an objective-type inverted microscope (Ti-E; Nikon) fitted with a 60×, NA 1.49 TIRF microscopy lens (Nikon) and controlled by iQ software (Andor Technology). While imaging, Dox (10 μM) was added to induce the DR module dissociation from the plasma membrane. To test a reversibility of the DR module upon Dox removal, cells were treated with 10 μM Dox for 10 min, washed and incubated in the culture media for 2 h. Together with the control samples treated with or without Dox, the cell samples were fixed with 4% paraformaldehyde (PFA) in PBS, and the EGFP fluorescence was visualized by a laser scanning confocal microscope (LSM 710; Zeiss) or Leica SP8 Gated STED 3× super resolution microscope. Changes in the redistribution of fluorescence between the plasma membrane profile and the cytoplasm (Figures 2A, 2B, and 2E by confocal microscopy) and in the plasma membrane fluorescence (Figures 2C and 2D by TIRF microscopy) were analyzed using Fiji^67^ (http://fiji.sc).

The Stimulated Emission Depletion (STED) microscopy images were acquired on a Leica SP8 Gated STED 3× super-resolution microscope equipped with a 100× objective (NA 1.4) with a commercial setting. Images were acquired using the Leica LAS-X software, with 512- or 1,024-pixel square fields with 22.7-nm pixel size. Images were edited and analyzed using Fiji (http://fiji.sc) in an experimental condition-blind manner. Each group has 292.4 ± 30.4 to 446.3 ± 40.3 PSD-95 puncta per image. For the quantitative analysis of two-channel colocalization, each channel image was filtered with Gaussian blur, adjusted with a threshold (MaxEntropy method), and subjected to mask selections. The signal intensity from the GluA1 channel in the selected masks of the PSD-95 channel or the percentage of overlapped mask selection of the GluA1 channel within the PSD-95 channel were quantified. To estimate the synapse number of each image, we quantified clustered PSD-95 puncta with an area equal to or larger than 0.02 square microns as a proxy of the synapse number (see the Table below).

**Table:** 

	ExSYTE_No Dox	ExSYTE_Dox	H64A_No Dox	H64A_Dox

Image number	21	28	22	33
Clustered PSD95 # / image	320.9 ± 42.1	292.4 ± 30.4	377.6 ± 38.5	446.3 ± 40.3
Mean ± SEM				

#### Fluorometry

CHO cells were transfected on a 96-well plate with DNA constructs expressing EGFP-fused candidate modules indicated in Figure S1D using FuGENE 6 (Promega). The next day, after PBS wash, the GFP signal from each well transfected with EGFP-fused candidate modules (Figure S1D) was measured at 25°C using a Victor2 microplate reader equipped with an F485 CW lamp Filter and an F535 CW emission filter (Perkin Elmer).

#### Electrophysiology using *Xenopus laevis* oocytes

Two-electrode voltage clamp (TEVC) recordings were performed as previously described.^63^ Briefly, ExSYTE (or ExSYTE^H64A^ control) cDNAs and HA-GluA1 were subcloned into pGEM-HE vector.^68^ The cRNAs were transcribed *in vitro* using T7 mMessage mMachine (Ambion) and injected (180 pg for ExSYTE and 100 pg for HA-GluA1) into defolliculated *Xenopus laevis* oocytes. TEVC analysis (at −70 mV) was performed 3 days after injection at room temperature. Glutamate (100 μM) was bath-applied with or without Dox (10 μM) in a recording solution containing 10 mM HEPES (pH 7.4), 90 mM NaCl, 1 mM KCl, and 1.5 mM CaCl_2_.

#### Western blot

Protein lysates from *Xenopus laevis* oocytes (Figure 3B) and HEK293 cells (Figure 4E) were prepared as previously described.^9,65^ Briefly, membrane pellets from oocytes were prepared by Dounce homogenization in a buffer containing 20 mM Tris (pH 8.0) and 5 mM EGTA and centrifugation at 20,000 × *g* for 30 s and solubilized with 0.3% Triton X-100 for 30 min at 4°C. After centrifugation at 20,000 × g for 5 min, the solubilized proteins were resolved by SDS-PAGE followed by western blot analysis. To solubilize proteins from HEK293 cells, cells were washed in PBS and lysed in a buffer containing 40 mM Tris (pH 7.4) and 1% SDS. After brief sonication to avoid viscosity and centrifugation at 20,000 × *g* for 5 min, the solubilized proteins were used for western blot analysis.

#### Electrophysiology in primary hippocampal neurons

Primary hippocampal neurons at DIV3–10 were infected with the AAV-ExSYTE (or ExSYTE^H64A^). Cultured neurons at DIV14–19 were transferred to a recording chamber continuously perfused with a solution saturated with 95% O_2_/5% CO_2_, and containing (in mM): 125 NaCl, 2.4 KCl, 2 CaCl_2_, 1 MgCl_2_, 25 NaHCO_3_, 1.2 NaH_2_PO_4_ and 25 Dextrose (301 mOsm, pH 7.4 adjusted with NaOH). Patch pipettes with tip resistances between 10–15 MΩ were filled with internal solution containing (in mM): 145 CsCl, 5 NaCl, 10 HEPES, 5 EGTA, 5 QX-314, 4 Mg-ATP and 0.3 Na-GTP (271 mOsm, pH 7.2 adjusted with CsOH). Miniature excitatory postsynaptic currents (mEPSCs) were monitored at room temperature in whole-cell configuration using a Multiclamp 700B amplifier (Axon Instruments) at a holding potential of −70 mV, and in the presence of picrotoxin (100 μM) and tetrodotoxin (1 μM). After recording for 3 min as ‘No Dox’, Dox (10 μM) was added and the recording continued for 11–17 min. Recordings with series resistances > 30 MΩ were rejected. Signals were filtered at 3 kHz and sampled at 25 kHz. Offline analysis was performed using Mini Analysis (http://www.synaptosoft.com, Decatur, GA, USA), with a threshold > 5 pA, a rise time between > 0.1 ms, decay time > 1 ms, and a half-width of ≥ ms.

#### AAV production

Adeno-associated virus (AAV) was generated using AAV-DJ Helper Free system (Cell Biolabs) as we published previously.^9^ To express ExSYTE selectively in the ‘Subthreshold fear conditioning’ neuronal population, the ExSYTE cassette was located in a reverse direction with two lox2272 and loxP loci (AAV-FLEX-ExSYTE) as illustrated in Figure 4D. The ExSYTE or ExSYTE^H64A^ fused with the P2A peptide-EGFP was cloned into the modified pAAV-MCS and transfected with pAAV-DJ and pHelper into 293AAV cells (Cell Biolabs). Transfected cells were lysed, and AAVs were purified with HiTrap heparin columns (GE Health) as previously described.^69^ AAV-EF1α-mCherry-IRES-Cre was purchased from Addgene (Cat. #55632-AAV8).

#### Virus injection

The six- to eight-week-old C57BL/6 mice were anesthetized with 1.5–3.0% isoflurane and placed in a stereotaxic apparatus (Kopf Instruments). Subcutaneous injections of ketoprofen (5 mg/kg body weight) were administered for 3 consecutive days beginning 1 day before the operation to prevent inflammation. The skull was exposed over the amygdala based on stereotactic coordinates. Then, 2 μl of AAV was bilaterally injected in the basolateral amygdala (BLA) and central amygdala (CeA) using a glass pipette (tip diameter 8~10 μm) at a rate of 200 nl/min using a syringe pump (Micro4; World Precision Instruments, Sarasota, FL). The injection site was standardized among animals by using stereotaxic coordinates (ML = ±3.0, AP = −1.5, DV = +4.5 for BLA and ML = ±2.25, AP = −1.0, DV = +4.5 for CeA) from bregma. To express oChIEF in the MGN, 0.5 μl of AAV was bilaterally injected using stereotaxic coordinates (ML = ±1.96, AP = −3.16, DV = +3.00) from bregma at a rate of 50 nl/min. At the end of injections, we waited 10 min before retracting the pipette.

#### Acute brain slice preparation

Mice were deeply anesthetized with isoflurane before decapitation. The brains were removed and rapidly transferred in an ice-cold dissection buffer maintained in 5% CO_2_/95% O_2_ and containing (in mM): 25 NaHCO_3_, 1.25 NaH_2_PO_4_, 2.5 KCl, 0.5 CaCl_2_, 7 MgCl_2_, 25 D-glucose, 110 choline chloride, 11.6 ascorbic acid, 3.1 pyruvic acid. Coronal brain slices (300 μm thick) were cut in ice-cold dissection buffer using a VT-1200S microslicer (Leica Microsystems Co.). Slices were transferred and incubated for 30 min at 32°C in the extracellular artificial cerebrospinal (ACSF) solution containing (in mM): 124 NaCl, 2.5 KCl, 26 NaHCO_3_, 1 NaH_2_PO_4_, 10 D-glucose, 1.3 MgSO_4_, 2.5 CaCl_2_. These slices were then kept at room temperature (22°C–25°C) for at least 30 min before recording. All solutions were equilibrated with 95% O_2_ and 5% CO_2_ (pH 7.4).

#### Optogenetics in acute brain slices

All experiments were performed in a submersion-type recording chamber perfused at ~2 ml min^−1^ with ACSF supplemented with the GABA_A_ receptor antagonist picrotoxin (100 μM). For On-Dox mice, Dox (10 μM) was added to the recovering and recording chambers. Slices were visualized using infrared differential interference contrast and GFP-expressing LA neurons were visualized using fluorescence video microscopy on a Nikon eclipse E600FN microscope. Patch-type pipette electrodes (~3–5 MΩ) were filled with intracellular solution containing (in mM): 115 cesium methanesulfonate, 20 CsCl, 10 HEPES, 2.5 MgCl_2_, 4 MgATP, 0.4 Na_3_GTP, 10 sodium phosphocreatine and 0.6 EGTA (pH 7.2, 280–290 mOsm). Whole-cell patch-clamp recordings using a Multiclamp 700A amplifier (Axon Instruments) were obtained from visually identified LA neurons clamped at −70 mV. Series resistance (~7–30 MΩ) was monitored throughout all experiments with a −5 mV, 80 ms voltage step, and cells that exhibited a significant change in series resistance (>20%) were excluded from analysis.

Light pulses of 0.5–2.0 ms, were used to evoke EPSCs driven by the ChIEF-expressing axons originating from the MGN. The light source was a high-power LED system (SOLIS-3C; ThorLabs Inc.) collimated and delivered through the microscope objective (40×, 0.8 NA). Synaptic responses were acquired at 5 kHz, filtered at 2.4 kHz, and analyzed using custom made software for IgorPro (Wavemetrics Inc.). LA neurons were recorded at holding potentials of −70 mV (for AMPAR-EPSCs) and +40 mV (for NMDAR-EPSCs). The AMPA/NMDA ratio was calculated as the ratio of peak current at −70 mV to the current at 30 ms after light stimulation onset at +40 mV (off-peak). Currents were monitored for 5 min and the quantification was made on the last 2.5 min of recording. To avoid wash-out in the pairing protocol experiments, a 2–4-min baseline was obtained and the magnitude of LTP was determined by comparing with responses 35–−40 min after LTP induction. Statistical analysis was performed using OriginPro software (OriginLab).

#### Histology

Mice were deeply anesthetized and transcardially perfused with 4% PFA in PBS (pH 7.4). Brains were fixed overnight in 4% PFA, and 40 μm thick coronal sections were cut on a vibratome at 4°C. Free floating sections were washed in PBS and then incubated for 45 min in 5% normal goat serum and 0.3% Triton X-100 in PBS. Slices were incubated overnight at room temperature with PBS containing 3% normal goat serum, 0.1% Triton X-100, and primary antibodies: guinea pig polyclonal anti-GFP (1:1,000) and mouse monoclonal anti-NeuN (1:1,000). Sections were then washed and incubated with secondary antibodies (Alexa Fluor 488 goat anti-guinea pig IgG and Alexa Fluor 546 goat anti-mouse IgG from Invitrogen) for 2 h at room temperature. Then, sections were washed in PBS and mounted on microscope slides with Fluoromount-G (Thermo Fisher Scientific). Confocal fluorescence images were acquired on a laser scanning confocal microscope (LSM 710; Zeiss) equipped with a 20× (NA 0.8) objective. Serial Z-stack images were converted to a single image and analyzed for cell counting using Fiji^67^ (http://fiji.sc). The image tiling approach was applied to Figures 4, S4, and S6, the tiling images were taken with 10.0 % overlap and the Meander online stitching (Zeiss ZEN Microscopy software). A region of interest centered in the BLA and CeA (manually defined based on NeuN staining) was used for counting numbers of the EGFP^+^ cells over the NeuN^+^ cells.

#### Behavioral experiments

All mice were housed on a 12 h light-dark cycle with food and water *ad libitum*. All experiments used approximately equal numbers of eight- to eleven-week-old littermates at the time of testing. To achieve the ‘Subthreshold fear conditioning’ that is sufficient to induce endogenous *c-Fos* activity but not cued fear memory, the foot shock intensity was modified to a mild level (0.15 mA) from the original protocol to test cued fear memory.^9^ The *c-Fos*-dependent targeted recombination in active populations (TRAP) of AAV-FLEX-ExSYTE 24 h after Tam administration (150 mg/kg body weight)^32^ was induced by delivering six times of 0.15 mA foot shocks (2 s) coupled with a tone cue (2.8 kHz, 90 dB, 20 s). This TRAP paradigm was sufficient to trigger the *c-Fos*-dependent ExSYTE expression in the BLA and CeA (Figures 4H, 4I, S4B, and S4C), but not to evoke cued fear memory (Figure 4G). After 1~2 weeks for protein expression and surgery recovery, freezing behavior to the tone used for the TRAP (2.8 kHz, 90 dB, 3 min) was monitored before (No Dox) and a day after Dox administration (On Dox) as well as 7 days after Dox withdrawal (Off Dox). Prior to each test, the mouse cages were relocated to the testing room and accommodated for at least 30 min. Dox was administrated by drinking water (2 mg/ml) containing 1% sucrose to mask the bitter taste of Dox.^70^ To avoid the effects of contextual cues, the behavior chambers were modified for each test. Every session was filmed using a video camera, and freezing behavior (defined as complete lack of movement between every 0.5-s frame of videos) was analyzed blindly to genotype. In addition, no obvious difference in body weight was observed. Statistical analysis was performed using GraphPad Prism 9 software.

### QUANTIFICATION AND STATISTICAL ANALYSIS

Quantification and statistical details of experiments, including the statistical tests used, exact value of n, what n represents (e.g., number of animals, number of cells, etc.) can be found (e.g., in the figure legends, figures, Results, Method Details section, etc.). All data are given as mean ± SEM. Statistical analysis was done with GraphPad Prism version 9. The normality of distributions was assessed using the Shapiro-Wilk test. In normal distributions, unpaired or paired Student’s t- est, one-way or two-way ANOVA with post hoc Tukey’s test were used to assess between-group and within-group differences. The non-parametric paired sample Wilcoxon signed rank test and Mann-Whitney U test and Kruskal-Wallis test with Dunn’s multiple comparisons test were used in non-normal distributions. Statistical significance was set to p < 0.05, and statistically significant differences are indicated as follows: *p < 0.05, **p < 0.01, and ***p < 0.001.

## Supplementary Material

1

## Figures and Tables

**KEY RESOURCES TABLE:** 

REAGENT or RESOURCE	SOURCE	IDENTIFIER

Antibodies		

Rabbit polyclonal anti-GluA1	Sumioka et al.^27^	N/A
Guinea pig polyclonal anti-GFP	Tomita et al.^58^	N/A
Guinea pig polyclonal anti-TARPγ-8	Sumioka et al.^27^	N/A
Mouse monoclonal anti-NeuN (clone A60)	Millipore	Cat# MAB377; RRID: AB_2298772
Mouse monoclonal anti-Actin (clone C4)	Millipore	Cat# MAB1501; RRID: AB_2223041
Goat anti-Guinea pig IgG, Alexa Fluor 488	Invitrogen	Cat# A-11073; RRID: AB_142018
Goat anti-Mouse IgG, Alexa Fluor 546	Invitrogen	Cat# A-11030; RRID: AB_144695

Bacterial and virus strains		

pAAV-Syn-ExSYTE	This paper	N/A
pAAV-Syn-ExSYTE^H64A^	This paper	N/A
pAAV-Syn-ExSYTE-P2A-EGFP-DTE	This paper	N/A
pAAV-Syn-FLEX-ExSYTE	This paper	N/A
pAAV-Syn-FLEX-ExSYTE^H64A^	This paper	N/A
pAAV-oChIEF-tdTomato	Lin et al.^32^	Addgene Plasmid #50977; RRID: Addgene_50977
pAAV-hSyn-DIO-EGFP	Laboratory of Bryan Roth	Addgene Plasmid #50457; RRID: Addgene_50457
AAV-EF1 α-mCherry-IRES-Cre	Fenno et al.^38^	Addgene AAV8; 55632-AAV8

Chemicals, peptides, and recombinant proteins		

FuGENE 6 transfection reagent	Promega	Cat# E2691
Doxycycline	Clontech	Cat# 631311
Tamoxifen	Sigma	Cat# T5648; CAS: 10540–29-1
TOTO-3 iodide (642/660)	Invitrogen	Cat# T3604
QX-314	Abcam	Cat# ab120118; CAS: 5369-03-9
Picrotoxin	Abcam	Cat# ab120315; CAS: 124-87-8
Tetrodotoxin	Cayman	Cat# 14963; CAS: 4368-28-9
Picrotoxin	Sigma-Aldrich	Cat# P1675; CAS: 124-87-8
HEPES	Sigma-Aldrich	H4034
NaCl	Sigma-Aldrich	S3014
KCl	JT baker	3040-01
CaCl_2_	Sigma-Aldrich	C4901
MgCl_2_	Sigma-Aldrich	M8266
Paraformaldehyde	Sigma-Aldrich	P6148
L-Glutamic acid	Sigma-Aldrich	G5889
Phosphate-buffered saline (PBS)	Gibco	14190144
Trizma	Sigma-Aldrich	T6066
Sodium dodecyl sulfate (SDS)	americanBIO	AB01920
Poly-D-lysine	Sigma-Aldrich	Cat# P6407
Papain	Sigma-Aldrich	P4762
NaHCO_3_	JT baker	3506-01
NaH_2_PO_4_	JT baker	3818-01
Dextrose	Sigma-Aldrich	G5767
NaOH	Fisher	SS255-1
CsCl	Sigma-Aldrich	C-3011
EGTA	Sigma-Aldrich	E4378
Mg-ATP	Sigma-Aldrich	A9187
Na-GTP	Sigma-Aldrich	G8877
CsOH	Sigma-Aldrich	C8518
Isoflurane	covetrus	11695-6777-2
Choline chloride	Sigma-Aldrich	C1879
Ascorbic acid	JT baker	B581-05
Sodium pyruvate	Sigma-Aldrich	P5280
MgSO_4_	Sigma-Aldrich	M5921
Cesium methanesulfonate	Sigma-Aldrich	C1426
Sodium phosphocreatine	Sigma-Aldrich	P7936
Normal goat serum	Vector Laboratories	Cat# S-1000
Triton X-100	Sigma	T8787
Fluoromount-G mounting medium	Thermo Fisher Scientific	Cat# 00-4958-02

Critical commercial assays		

mMESSAGE mMACHINE T7 transcription kit	Invitrogen	Cat# AM1344

Experimental models: Cell lines		

HeLa cells	ATCC	Cat# CCL-2; RRID: CVCL_0030
HEK293 cells	ATCC	Cat# CRL-1573; RRID: CVCL_0045
CHO cells	ATCC	Cat# CCL-61; RRID: CVCL_0214
293AAV cells	Cell Biolabs	Cat# AAV-100; RRID: CVCL_KA64

Experimental models: Organisms/strains		

C57BL/6J mice	The Jackson Laboratory	Stock# 000664; RRID: IMSR_JAX:000664
FosTRAP mice: B6.129(Cg)-Fos^tm1.1(cre/ERT2)Luo^/J	The Jackson Laboratory	Stock# 021882; RRID: IMSR_JAX:021882
*Xenopus laevis* oocytes	Nasco	Adult female

Oligonucleotides		

Primers for DNA constructs, see Table S1	This paper	N/A

Recombinant DNA		

pCAGTetRnls	Zafarana et al.^19^	Addgene Plasmid #26599; RRID: Addgene_26599
pTet-Off Advanced Vector	Clontech	Cat# 631070
pEGFP-C2-TetR	This paper	N/A
pEGFP-C2-KRφ-TetR	This paper	N/A
pEGFP-C2-TetR-KRφ-TetR	This paper	N/A
pEGFP-C2-TetR-KRφ(-K)-TetR	This paper	N/A
pEGFP-C2-TetR-KRφ(-LK)-TetR	This paper	N/A
pEGFP-C2-TetR-KRφ(-KLK)-TetR	This paper	N/A
pEGFP-C2-DR	This paper	N/A
pmCherry-C1-DR^H64A^	This paper	N/A
pGEM-HE-ExSYTE	This paper	N/A
pGEM-HE-ExSYTE^H64A^	This paper	N/A
pGEM-HE-HA-GluA1	Kim et al.^60^	N/A
pGW1-ExSYTE-EGFP-DTE	This paper	N/A
pGW1-ExSYTE-EGFP	This paper	N/A
pGW1-ExSYTE-P2A-EGFP	This paper	N/A
piRFP	Filonov et al.^28^	Addgene Plasmid #31857; RRID: Addgene_31857

Software and algorithms		

Volocity software	Perkin Elmer	https://www.perkinelmer.com/lab-products-and-services/resources/whats-new-volocity-6-3.html
iQ software	Andor Technology	https://andor.oxinst.com/products/iq-live-cell-imaging-software/
LabChart	ADInstruments	https://www.adinstruments.com/products/labchart/
Fiji	Schindelin et al.^62^	http://fiji.sc
Mini Analysis Program	Synaptosoft	http://www.synaptosoft.com/MiniAnalysis/
GraphPad Prism 9	GraphPad	https://www.graphpad.com/scientific-software/prism/
Video Freeze software	Med Associates	https://www.med-associates.com/product/video-fear-conditioning/
IgorPro	Wavemetrics	https://www.wavemetrics.com/
OriginPro 9	OriginLab	http://www.originlab.com/
Huygens STED deconvolution software	Scientific Volume Imaging	https://svi.nl/Huygens-STED-Software
Leica LAS-X software	Leica	Leica
Zeiss ZEN Microscopy software	Zeiss	Zeiss

Other		

Dulbecco's Modified Eagle Medium (DMEM)	Gibco	11965092
Fetal bovine serum	Gibco	10437028
F-12 medium	Gibco	11765054
UltraVIEW VoX spinning disk confocal microscope	Perkin Elmer	UltraVIEW VoX
14-bit EMCCD camera	Hamamatsu	C9100-50
Objective-type inverted TIRF microscope	Nikon	Ti-E
60x NA 1.49 TIRF microscopy lens	Nikon	MRD01691
Zeiss LSM 710 laser scanning confocal microscope	Zeiss	LSM 710
20x NA 0.8 objective lens	Zeiss	420650-9902-000
Leica SP8 Gated STED 3x super resolution microscope	Leica	SP8
HC PL APO 100x/1,40 OIL STED WHITE	Leica	11506378
Victor2 microplate reader	Perkin Elmer	Victor2
Neurobasal medium	Gibco	21103049
Glutamax	Gibco	35050079
B27 supplement	Gibco	17504044
Penicillin/Streptomycin	Gibco	15140122
Multiclamp 700B amplifier	Axon Instruments	MultiClamp 700B
AAV-DJ Helper Free system	Cell Biolabs	VPK-400-DJ
HiTrap heparin columns	GE Healthcare	Cat# 17-0406-01
Stereotaxic apparatus	Kopf Instruments	Model 940
Micro syringe pump	World Precision Instruments	Micro4
VT-1200S microslicer	Leica Microsystems Co.	VT-1200S
Nikon eclipse E600FN microscope	Nikon	E600FN
Multiclamp 700A amplifier	Axon Instruments	MultiClamp 700A
High-power LED system	ThorLabs Inc.	SOLIS-3C
40x 0.8 NA microscope objective	Nikon	MRF07420

## Highlights

- ExSYTE, a chemogenetic tool, can control protein-lipid electrostatic interactions
- Dissociation of the TARP cytoplasmic domain from plasma membranes mimics AMPAR-LTP
- A subthreshold conditioning induces c-Fos expression in the amygdala without freezing
- ExSYTE expression in activated amygdala neurons reversibly controls freezing
