# Supplementary material for: Chemogenetic regulation of the TARP-lipid interaction mimics LTP and reversibly modifies behavior
Source: Cell Rep. Author manuscript; Available in PMC 2023 Sep 27. (PMC10528344; doi:10.1016/j.celrep.2023.112826)
Supplement: 1 [file NIHMS1928226-supplement-1.pdf]

Cell Reports, Volume *42*

Supplemental information

Chemogenetic regulation of the TARP-lipid interaction mimics LTP and reversibly modifies behavior

Joongkyu Park, Coralie Berthoux, Erika Hoyos-Ramirez, Lili Shan, Megumi Morimoto-Tomita, Yixiang Wang, Pablo E. Castillo, and Susumu Tomita

Supplemental Information

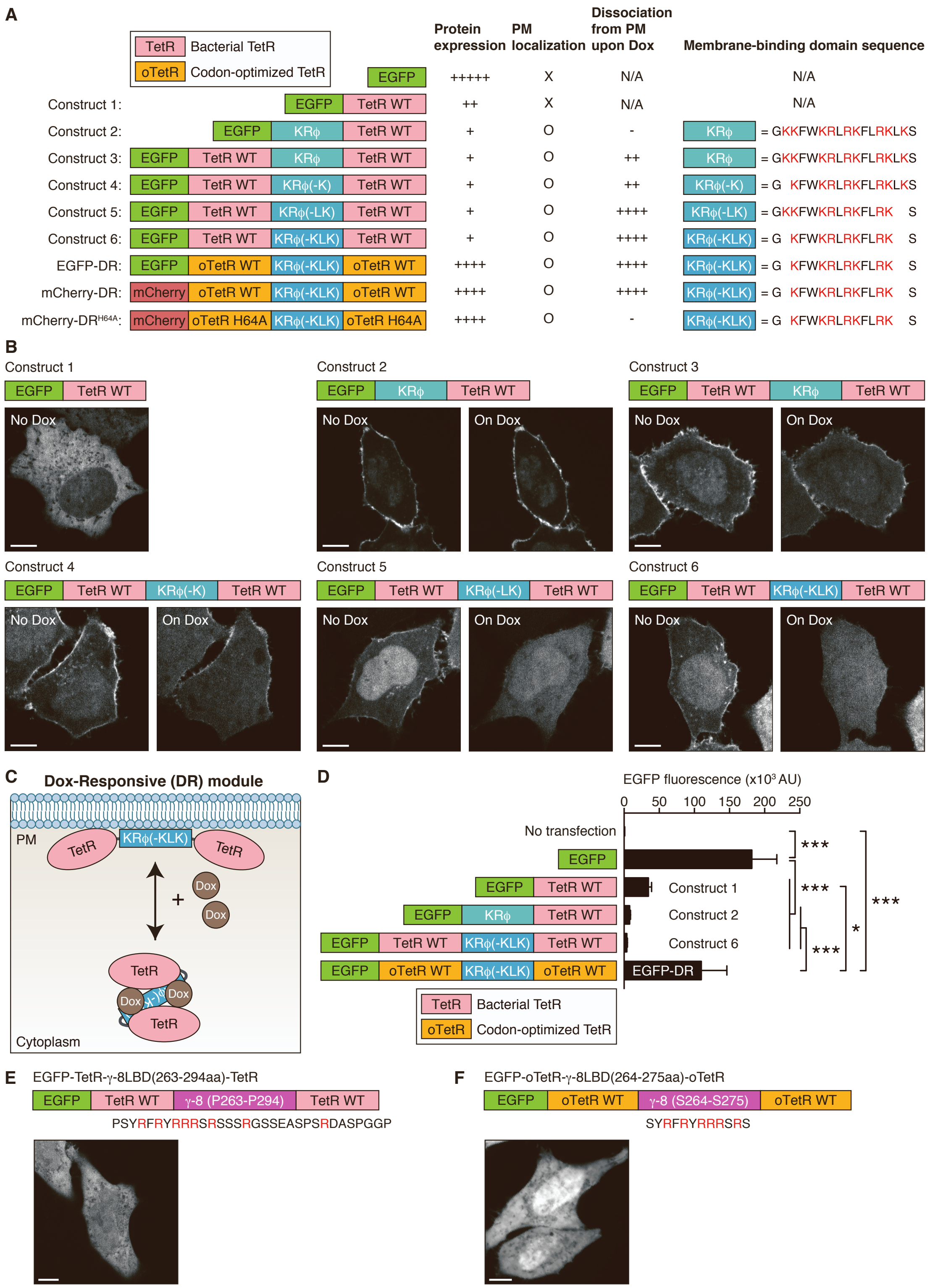

Figure S1. Development of a chemogenetic lipid-binding module (related to Figure 1)
(A) Schematic summary of the development of the Dox-Responsive lipid-binding module (DR). Each candidate module with EGFP or mCherry was examined for its protein expression, cellular localization to the plasma membrane, and Dox-dependent dissociation from the plasma membrane. The optimized DR contains a modified polybasic lipid-binding domain 'KRϕ(-KLK)' that is originated from a polybasic amphiphilic helix (KRϕ) (Roy et al., 2000; Yeung et al., 2006) and two of a codon-optimized tetracycline repressor (oTetR). Introduction of a single point mutation into the oTetR (H64A; alanine substitution at histidine 64 residue of oTetR) disables the Dox-binding (referred as 'DR^{H64A}').
(B) Representative micrographs showing the localization of each candidate module at the plasma membrane of transiently transfected HeLa cells before (No Dox) and after Dox treatment (On Dox). Scale bar, 10 μm. Addition of a polybasic lipid-binding domain (KRϕ) localized proteins at the plasma membranes (constructs 2, 3, 4, 5, 6), and having two TetRs dissociates proteins from the plasma membranes upon Dox application (constructs 3, 4, 5, and 6). To improve the response to Dox, we further modified KRϕ by reducing numbers of basic residues (constructs 4, 5, 6). Both constructs 5 and 6 showed the most robust responses to Dox.
(C) Putative model of the DR module showing that Dox binds to the TetRs and induces dimerization of two TetR proteins that mask the lipid-binding domain, KRϕ(-KLK), thereby dissociating the module from the plasma membrane (PM).
(D) Comparison of protein expression levels of each construct. Fluorometric analysis of the EGFP-fused candidate modules expressed in CHO cells transfected transiently. Whereas the constructs containing bacterial TetRs (constructs 1, 2 and 6) were expressed at a low level, the EGFP-DR construct containing a codon-optimized TetR (oTetR) was expressed at a substantially higher level.
(E, F) Distribution of the DR module mutant replacing KRϕ(-KLK) with the lipid binding domain (E) or the Arginine-rich domain (F) of TARPγ-8 (amino acid sequences are shown).
Data are shown as mean ± s.e.m. n = 5-15 cells, *P < 0.05, ***P < 0.001, one-way ANOVA followed by post hoc Tukey's multiple comparisons.

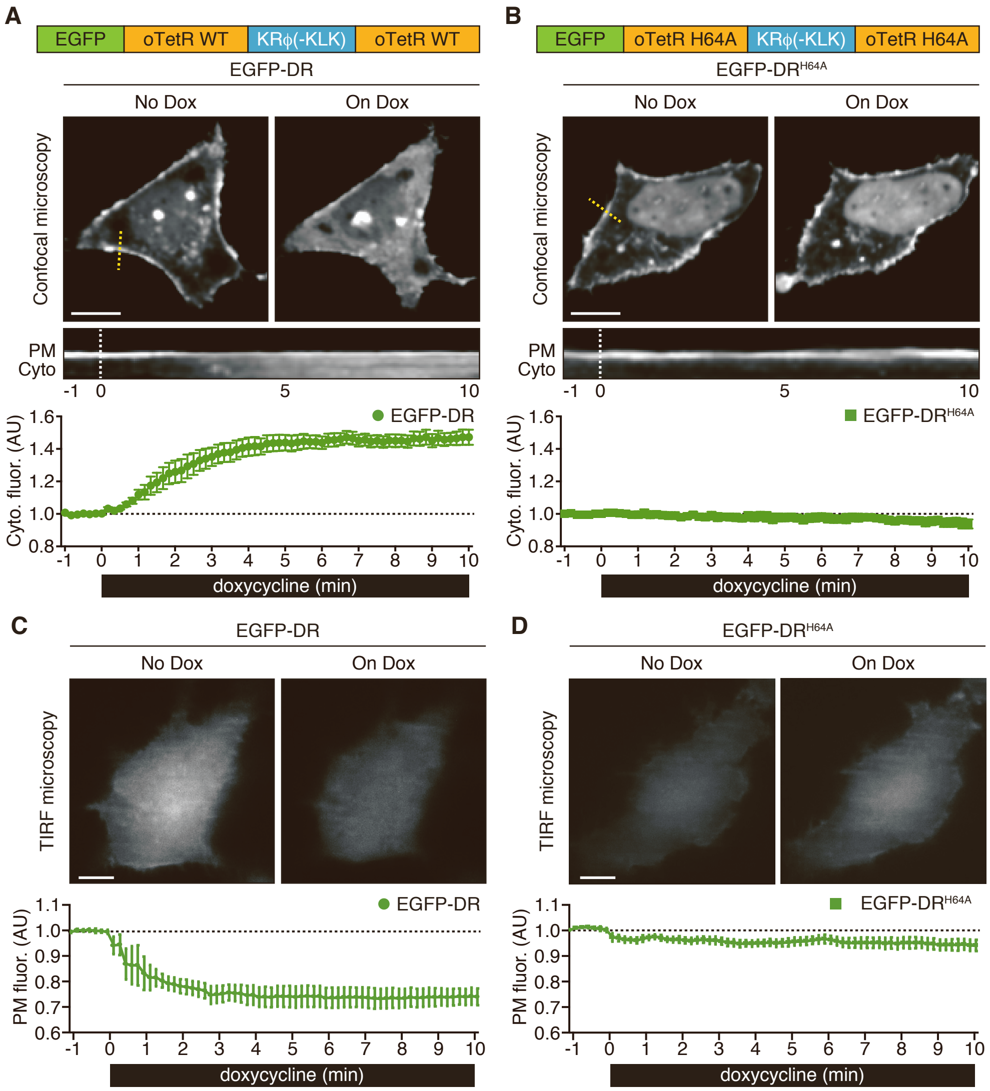

Figure S2. Distribution of EGFP-tagged chemogenetic lipid-binding modules (related to Figure 2)
Distribution of EGFP-fused DR and DR mutant (H64A) was examined in transiently transfected HeLa cells by spinning disc confocal (A and B) and TIRF (C and D). Both EGFP-DR (A and C) and EGFP-DR^{H64A} (B and D) localized at the plasma membrane (No Dox). Ten minutes after 10 µM Dox treatment (On Dox), EGFP-DR, but not EGFP-DR^{H64A}, showed increased cytoplasmic signal in spinning disc confocal (A and B) and reduced TIRF signal from the proximal plasma membrane (C and D). Representative images (top), kymographs (middle; from the dashed yellow lines in A and B) and quantification (bottom) of cytoplasmic fluorescence (A and B; *n* = 5-7 cells) and proximal plasma membrane fluorescence (C and D; *n* = 6 cells). Scale bar, 10 µm. Data are presented as mean ± s.e.m.

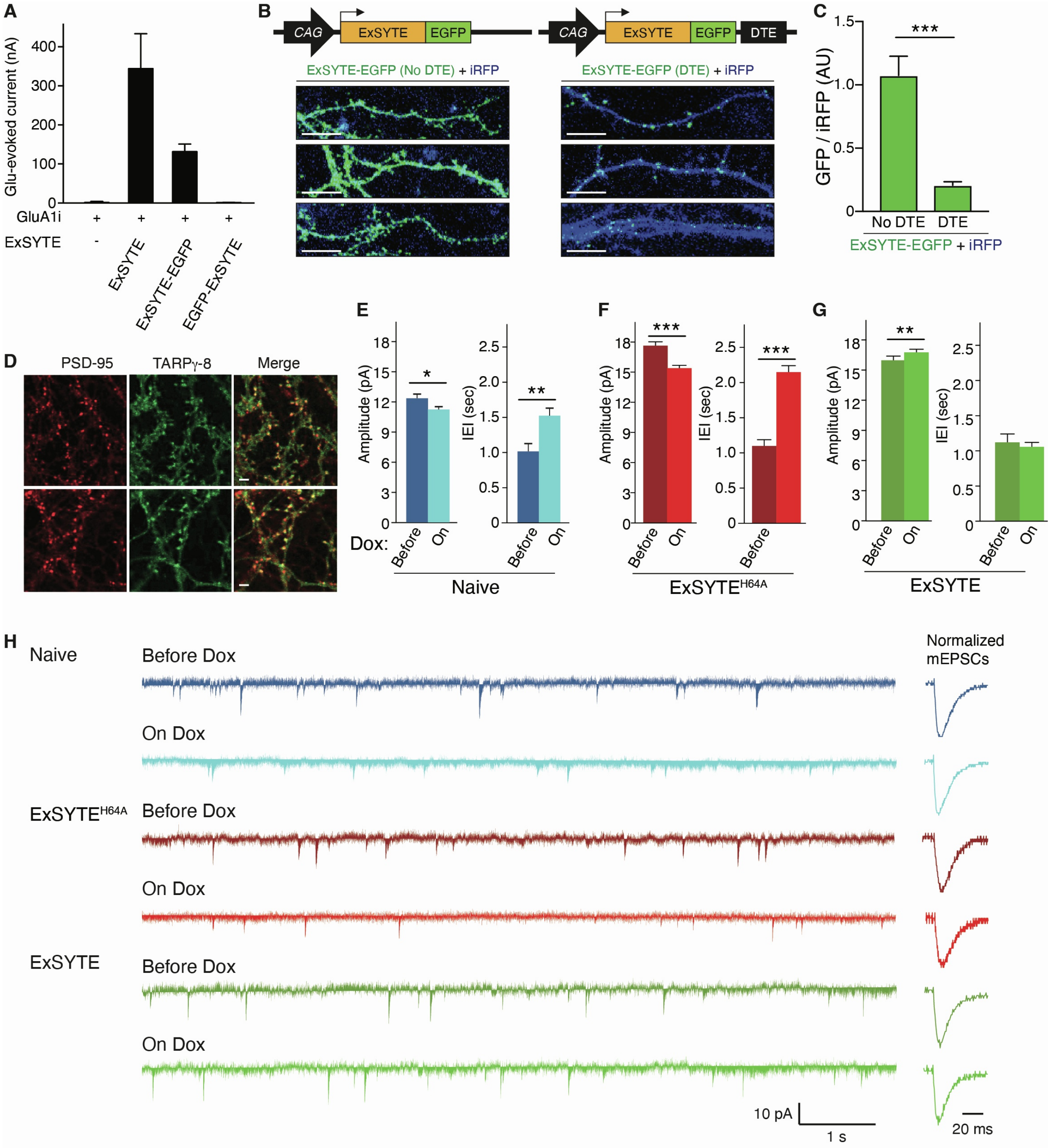
A
Glu-evoked current (nA)
400
300
200
100
0
GluA1i
ExSYTE
+
-
ExSYTE
ExSYTE-EGFP
EGFP-ExSYTE
B
CAG
ExSYTE
EGFP
DTE
ExSYTE-EGFP (No DTE) + iRFP
ExSYTE-EGFP (DTE) + iRFP
C
GFP / iRFP (AU)
1.5
1.0
0.5
0
***
No DTE
DTE
ExSYTE-EGFP + iRFP
D
PSD-95
TARPγ-8
Merge
E
Amplitude (pA)
IEI (sec)
Dox:
Before
On
Naive
F
ExSYTE^H64A
G
ExSYTE
H
Naive
Before Dox
On Dox
ExSYTE^H64A
Normalized mEPSCs
10 pA
1 s
20 ms

Figure S3. Further characterization of ExSYTE constructs (related to Figure 3)
(A) GluA1 flip cRNA (GluA1i, 100 pg) was co-injected with one of ExSYTE (100 pg), N-terminally EGFP-tagged ExSYTE (EGFP-ExSYTE, 144 pg), or C-terminally EGFP tagged ExSYTE (ExSYTE-EGFP, 144 pg) into *Xenopus laevis* oocytes. The amounts of ExSYTE cRNAs were adjusted by the length of cRNAs. Glutamate (Glu)-evoked currents (5 μM) with 50 μM cyclothiazide (CTZ) were monitored using two-electrode voltage clamp recording (V_h = -70 mV). Both ExSYTE and ExSYTE-EGFP enhanced glutamate-evoked currents robustly, whereas EGFP-ExSYTE did not (n = 6 oocytes).
(B) DTE-dependent expression of ExSYTE-EGFP. An EGFP-fused ExSYTE construct without or with an *Arc* mRNA DTE was co-transfected with an iRFP construct into primary hippocampal neurons at DIV14-15, and cells were imaged 40 hours later. Whereas ExSYTE-EGFP (green) without a DTE was diffusely expressed in neurons, ExSYTE-EGFP with DTE was detected as puncta on spines and dendrites. The iRFP (blue) was labeled in most spines and neurites as a control. Scale bar, 10 μm.
(C) Summary plot showing the intensity of ExSYTE-EGFP relative to iRFP intensity in spines and neurites without or with DTE (n = 15 dendrites).
(D) Primary hippocampal neurons were co-stained with anti PSD-95 and TARPγ-8 antibodies. TARPγ-8 localized at spines labeled with PSD-95. Scale bars, 2 μm.
(E-H) Summary plot of mEPSC amplitude and frequency in cultured neurons (E-G) and representative mEPSC traces (H). Dox reduced the mEPSC frequency of naïve (E) and the H64A expressing neurons (F), but did not alter the mEPSC frequency in the ExSYTE expressing neurons (G). Dox increased the mEPSC amplitude in neurons expressing ExSYTE, but not those expressing inactive $ExSYTE^{H64A}$ and naïve neurons (n = 252/493 for 3 naïve neurons, 827/1413 for 7 H64A neurons, 1026/2476 for 7 ExSYTE neurons).
Data are shown as mean ± s.e.m. *P < 0.05, **P < 0.01, ***P < 0.001; Mann-Whitney test (C, E, F, G).

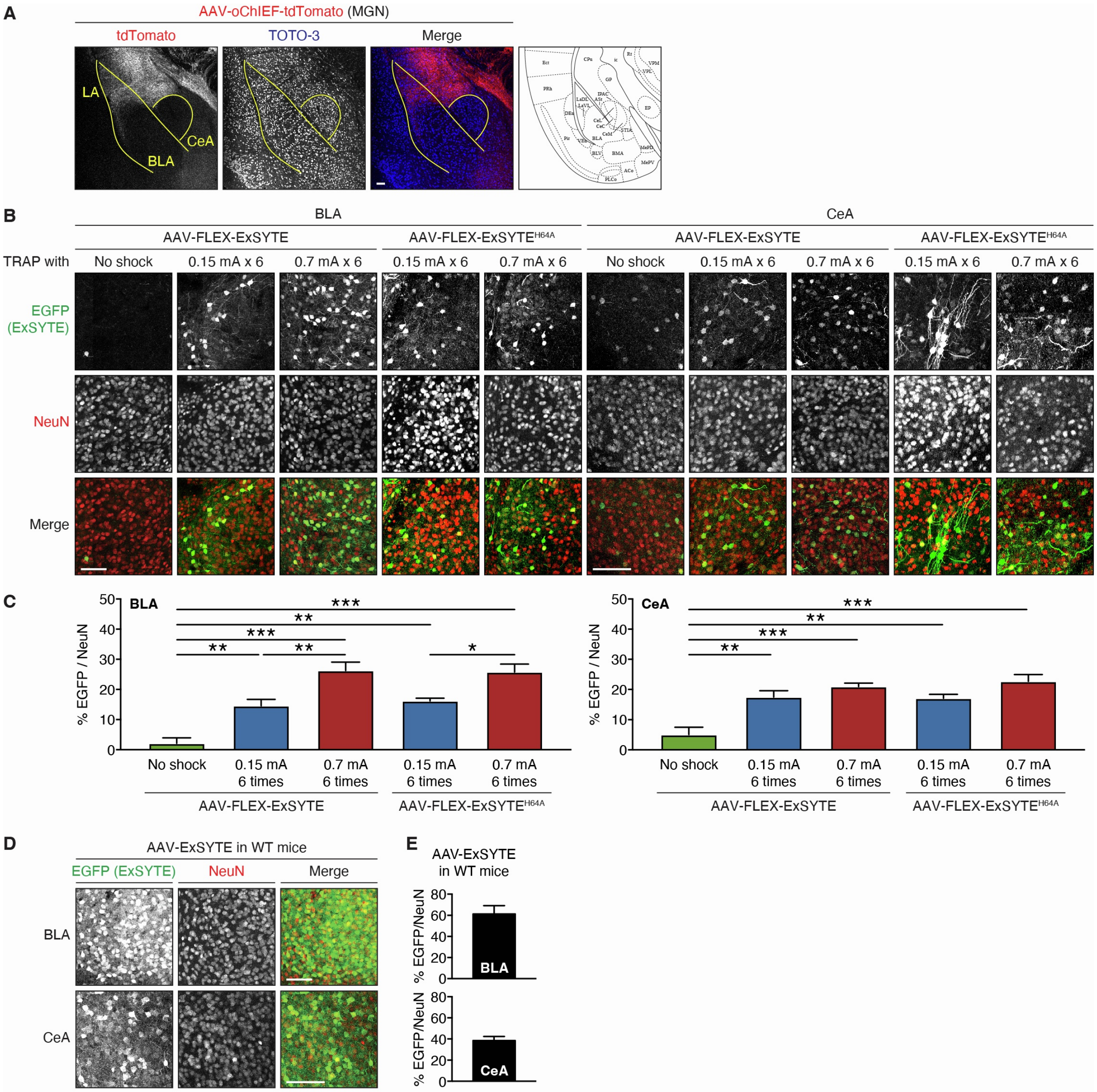

A
AAV-oChIEF-tdTomato (MGN)
tdTomato
TOTO-3
Merge
LA
CeA
BLA
B
BLA
CeA
AAV-FLEX-ExSYTE
AAV-FLEX-ExSYTE^H64A
TRAP with
No shock
0.15 mA x 6
0.7 mA x 6
EGFP (ExSYTE)
NeuN
Merge
C
BLA
CeA
% EGFP / NeuN
No shock
0.15 mA 6 times
0.7 mA 6 times
AAV-FLEX-ExSYTE
AAV-FLEX-ExSYTE^H64A
D
AAV-ExSYTE in WT mice
EGFP (ExSYTE)
NeuN
Merge
BLA
CeA
E
AAV-ExSYTE in WT mice
% EGFP/NeuN
BLA
CeA

Figure S4. Subthreshold fear conditioning induces *c-fos*-dependent ExSYTE expression (related to Figure 4)
(A) Representative images showing an expression of oChIEF-tdTomato in the MGN and its axonal projection to the LA. AAV-oChIEF-tdTomato was injected into the MGN, and fixed coronal brain sections were prepared 7 days later for confocal imaging. Images were taken as tiled images. The oChIEF-tdTomato expression in the MGN and axonal projection from the MGN to the LA were validated by tdTomato fluorescence (red) based on tissue morphology stained by a nuclear dye TOTO-3 (blue). The reference image was modified from "The Mouse Brain" atlas. Scale bar, 100 μm.
(B and C) ExSYTE (or $ExSYTE^{H64A}$) expression was examined in mice conditioned with the subthreshold US condition (0.15 mA, 6 times) and a conventional US condition (0.7 mA, 6 times). Mice were injected with AAV-FLEX-ExSYTE (or $ExSYTE^{H64A}$) -P2A-EGFP in the basolateral amygdala (BLA) and the central nucleus of the amygdala (CeA) followed by the procedure indicated in Figure 4F. One to two weeks after injection Tamoxifen (Tam; 150 mg/kg body weight) was injected intraperitoneally, and 24 hr later mice were subjected to cued fear conditioning with various intensity of US (foot shock) to examine expressions of EGFP/ExSYTE and EGFP/ExSYTE H64A. Images were taken as tiled images. Representative confocal micrographs (B) and quantification (C). Coronal sections from the mice were immunostained with anti-GFP (green) and anti-NeuN (red) antibodies (n =5-6 animals per group). Scale bar, 100 μm. These results identified a subthreshold US condition (0.15 mA, 6 times) that enables *c-fos* promoter-dependent EGFP/ExSYTE expression without forming cued fear memory (Figure 4G). A part of BLA result is displayed repeatedly as Figures 4H and 4I.
(D and E) AAV-ExSYTE was injected into the amygdala of WT mice (n =5 animals). Coronal sections from AAV-injected mice showing GFP and NeuN immunolabeling. Images were taken as tiled images. Representative confocal micrographs (D) and quantification (E) are shown. Scale bar, 100 μm.
Data are shown as mean ± s.e.m. *$P < 0.05$, **$P < 0.01$, ***$P < 0.001$, one-way ANOVA followed by post hoc Tukey's multiple comparisons.

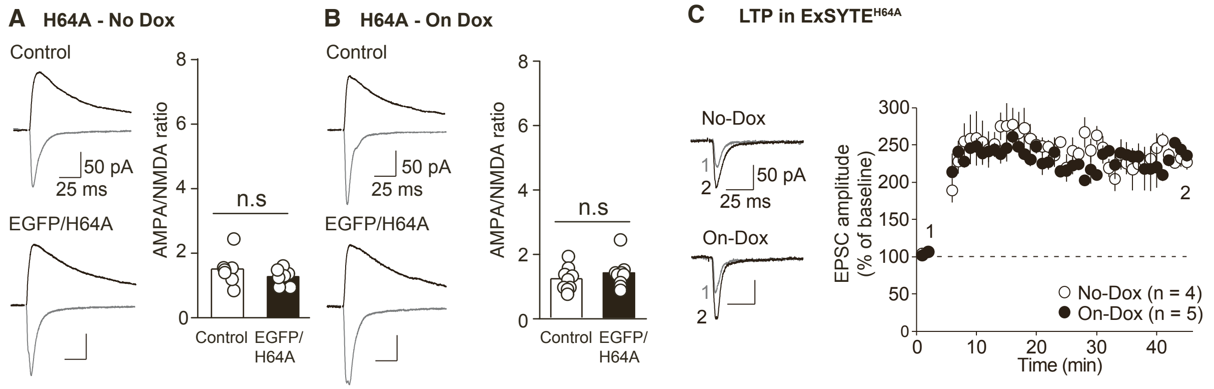

Figure S5. Inactive ExSYTE^{H64A} expression does not alter synaptic AMPAR-mediated transmission (related to Figure 5)

All procedures followed the scheme presented in Figure 5A except the notion that AAV-FLEX-ExSYTE^{H64A}-P2A-EGFP was injected in the amygdala instead of AAV-FLEX-ExSYTE-P2A-EGFP.

(A, B) AMPA/NMDA ratio does not differ between control and EGFP/ExSYTE^{H64A} cells before Dox administration (A, n = 7 cells from 4 animals each) and with 1 d Dox administration (B, n = 9 cells from 6 animals each).

(C) LTP was observed in EGFP/ExSYTE^{H64A} cells with No Dox (n = 5 cells from 4 animals) and On Dox (n = 4 cells from 3 animals). Representative averaged EPSCs (left) and summary plot (right).

Data are presented as mean ± s.e.m. n.s., non-significant, paired Student's *t*-test (A) and Mann-Whitney's U test (B).

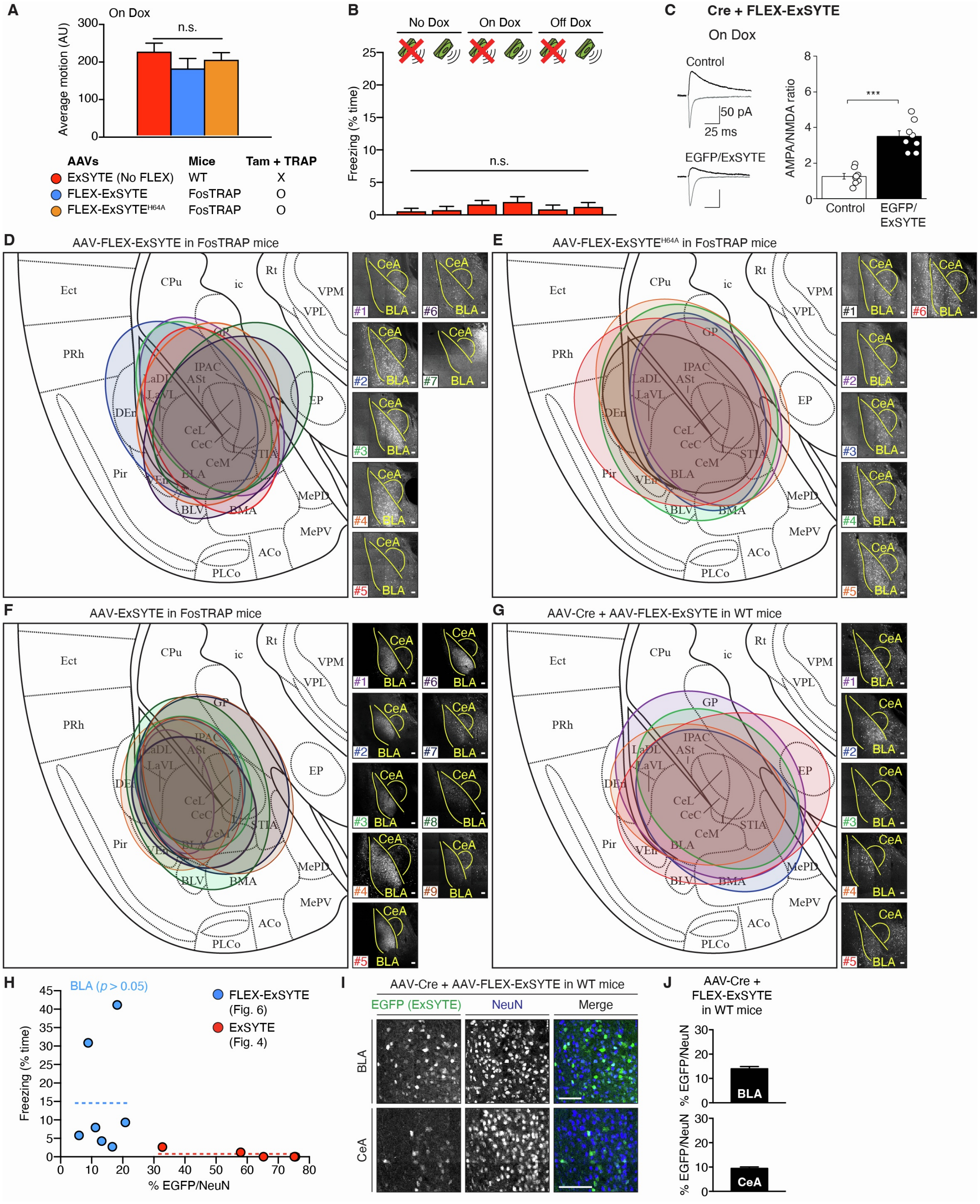
A
On Dox
Average motion (AU)
n.s.
AAVs
Mice
Tam + TRAP
ExSYTE (No FLEX)
WT
X
FLEX-ExSYTE
FosTRAP
O
FLEX-ExSYTE H64A
FosTRAP
O
B
No Dox
On Dox
Off Dox
Freezing (% time)
n.s.
C
Cre + FLEX-ExSYTE
On Dox
Control
50 pA
25 ms
EGFP/ExSYTE
AMPA/NMDA ratio
***
Control
EGFP/ ExSYTE
D
AAV-FLEX-ExSYTE in FosTRAP mice
E
AAV-FLEX-ExSYTE H64A in FosTRAP mice
F
AAV-ExSYTE in FosTRAP mice
G
AAV-Cre + AAV-FLEX-ExSYTE in WT mice
Ect
CPu
ic
Rt
VPM
VPL
PRh
GP
IPAC
LaDL
ASt
LaVL
EP
DEn
CeL
CeC
CeM
STIA
Pir
VEn
BLA
BLV
BMA
MePD
MePV
ACo
PLCo
CeA
H
BLA (p > 0.05)
FLEX-ExSYTE (Fig. 6)
ExSYTE (Fig. 4)
Freezing (% time)
% EGFP/NeuN
I
AAV-Cre + AAV-FLEX-ExSYTE in WT mice
EGFP (ExSYTE)
NeuN
Merge
BLA
CeA
J
AAV-Cre + FLEX-ExSYTE in WT mice
% EGFP/NeuN
BLA
CeA

Figure S6. Verification of EGFP/ExSYTE expression (related to Figure 6)
(A) General locomotion activity was assessed by measuring the averaged motion (arbitrary units) in the On Dox session. Red, WT mice injected with AAV-ExSYTE-P2A-EGFP in the amygdala; blue and orange, FosTRAP mice injected with AAV-FLEX-ExSYTE-P2A-EGFP or AAV-FLEX-ExSYTE^{H64A}-P2A-EGFP in the amygdala and subjected to a subthreshold conditioning (related to Figures 6B-6D).
(B) Mice did not freeze without or with a tone (2.8 kHz, 90 dB) regardless of Dox administration (n = 5 animals). n.s., non-significant, one-way ANOVA.
(C) Optically-evoked AMPAR- and NMDAR-EPSCs at the MGN-LA synapses were measured after 1 d Dox administration (On Dox) in EGFP-positive (EGFP/ExSYTE) or EGFP-negative (control) cells of acute brain slices injected with AAV-FLEX-ExSYTE together with AAV.EF1α promoter mCherry-IRES-Cre (AAV-Cre). AMPA/NMDA ratio was increased in EGFP/ExSYTE cells (n = 8 cells from 5 mice) as compared to controls (n = 8 cells from 5 mice).
(D-G) Histologically verified expression of ExSYTE in mice (as indicated) injected with AAV-FLEX-ExSYTE (D), AAV-FLEX-ExSYTE^{H64A} (E), AAV-ExSYTE (non-FLEX) (F), or AAV-FLEX-ExSYTE together with AAV-Cre (G). The AAVs were injected (as indicated) into the amygdala of FosTRAP (or WT) mice. After behavioral tests, coronal sections from the mice were immunostained with anti-GFP antibody. Images were taken as tiled images. The areas displaying EGFP/ExSYTE-positive cells from each mouse are highlighted by distinct colors (left) based on individual staining data (right). Scale bars, 100 μm.
(H) Freezing of AAV-FLEX-ExSYTE mice (blue) and AAV-ExSYTE mice (red) is plotted against the fraction of infected cells in the BLA (same data as in Figures 4 and 6). Each dot represents values from a single animal. Dotted lines indicate average freezing in each group.
(I and J) AAV-FLEX-ExSYTE and AAV-Cre were co-injected into the amygdala of WT mice. One to two weeks later, coronal sections from AAV-injected mice were immunostained with anti-GFP and anti-NeuN antibodies. Images were taken as tiled images. Representative confocal micrographs (I) and quantification (J) are shown. EGFP signal derived from ExSYTE-P2A-EGFP (green) was detected in 14% and 9% of NeuN-positive cells (blue) in the BLA and CeA, respectively (n = 5 animals). Scale bar, 100 μm.
Data are shown as mean ± s.e.m. n.s., non-significant, ***P < 0.001, unpaired t-test (C). The reference image was modified from "The Mouse Brain" atlas.

Table S1. Primers used for general, mutagenesis, and overlap extension PCR to generate each construct.

Construct		Primer
pEGFP-C2-TetR	Fwd	5'-GCACA**CTCGAG***CATGTCTAGATTAGATAAAAGTAAAG* -3'<br>(XhoI in bold)
	Rev	5'-GCACA**GAATTC**TTA*AGACCCACTTTCACATTTAAGTTG* -3'<br>(EcoRI in bold)
pEGFP-C2-KRϕ-TetR	Fwd	5'-GCACA**CTCGAG**CGGCAAGAAATTTTGGAAGAGGCTGAGAAAATTCCTCCGGAAGCTGAAAAGC*ATGTCTAGATTAGATAAAAGTAAAG* -3'<br>(XhoI in bold)
	Rev	5'-GCACA**GAATTC**TTA*AGACCCACTTTCACATTTAAGTTG* -3'<br>(EcoRI in bold)
pEGFP-C2-TetR-KRϕ-TetR	Fwd #1	5'-GCACA**CTCGAG**CGAGCGCAGCCGCGAGGCA*ATGTCTAGATTAGATAAAAGTAAAG* -3'<br>(XhoI in bold)
	Rev #1	5'-GAGGAATTTTCTCAGCCTCTTCCAAAATTTCTTGCC*AGACCCACTTTCACATTTAAGTTG* -3'
	Fwd #2	5'-AAGAGGCTGAGAAAATTCCTCCGGAAGCTGAAAAGC*ATGTCTAGATTAGATAAAAGTAAA* -3'
	Rev #2	5'-GCA**GAATTC**CTACACGGGCGTGGTTTTCCTGTTGAG*AGACCCACTTTCACATTTAAGTTG* -3'<br>(EcoRI in bold)
pEGFP-C2-TetR-KRϕ(-K)-TetR	Fwd	5'-*GTGAAAGTGGGTCT* GGCAAATTTTGGAAGAGG-3'
pEGFP-C2-TetR-KRϕ(-KLK)-TetR	Rev	5'-CCTCTTCCAAAATTTGCC*AGACCCACTTTCAC* -3'
pEGFP-C2-TetR-KRϕ(-LK)-TetR<br>pEGFP-C2-TetR-KRϕ(-KLK)-TetR	Fwd	5'-GAGAAAATTCCTCCGGAAGAGC*ATGTCTAGATTAGATAAAAGT* -3'
	Rev	5'-*ACTTTTATCTAATCTAGACAT* GCTCTTCCGGAGGAATTTTCTC-3'
pEGFP-C2-DR<br>(= oTetR-KRϕ<br>[-KLK]-oTetR)	Fwd #1	5'-GCA**CTCGAG**CGAGCGCAGCCGCGAGGCA*ATGTCTAGACTGGACAAGAGCAA* -3'<br>(XhoI in bold)
	Rev #1	5'-CCGGAGGAATTTTCTCAGCCTCTTCCAAAATTTGCC*GCTGCCGCTTTCGCACTTTAGCTG* -3'
	Fwd #2	5'-GGAAGAGGCTGAGAAAATTCCTCCGGAAGAGC*ATGTCTAGACTGGACAAGAGCAA* -3'
	Rev #2	5'-GCA**GAATTC**CTATACGGGCGTGGTCCGGCGGTTAAGCTTACTAGT*GCTGCCGCTTTCGCACTTTAGCTG* -3'<br>(EcoRI in bold)
pmCherry-C1-DR^{H64A}	Fwd	5'-GATGCTGGACAGGCATGCTACCCACTTCTGCCCC-3'<br>(H64A in red)
	Rev	5'-GGGGCAGAAGTGGGTAGCATGCCTGTCCAGCATC-3'<br>(H64A in red)
for P2A-EGFP	Fwd #1	5'-GCA**CTGCAG**CGCCCGCGCCCGGGACC-3'<br>(PstI in bold)
	Rev #1	5'-CAGGCTGAAGTTAGTAGCTCCGCTTCCCACGGGCGTGGTTTTCCTG-3'
	Fwd #2	5'-CAGGAAAACCACGCCCGTGGGAAGCGGAGCTACTAACTTCAGCCTG-3'
	Rev #2	5'-GCA**TCTAGA**CTACTTGTACAGCTCGTCCATGC-3'<br>(XbaI in bold)
for EGFP tagging	Fwd	5'-GAAGCAGGCTGGAGACGTGGAGATGGTGAGCAAGGGCGAGGAG-3'
	Rev	5'-CTCCTCGCCCTTGCTCACCATCTCCACGTCTCCAGCCTGCTTC-3'
for DTE of rat<br>*Arc* mRNA	Fwd	5'-GCACA**TCTAGA**TCGGCTCCATGACTCAGCCATGCC-3'<br>(XbaI in bold)
	Rev	5'-GCACA**TCTAGA**AGACACGAGCAGTTACCAACACG-3'<br>(XbaI in bold)

(Enzyme sites are indicated in bold. Lipid-binding domains are underlined. The TetR sequence is indicated in italic.)
